# Multilevel genomic constraints shape nuclear tRNA gene organization in plants

**DOI:** 10.1111/tpj.71075

**Published:** 2026-08-04

**Authors:** Guillaume Hummel, David Pflieger, Valérie Cognat, Laurence Drouard, Alexandre Berr

**Affiliations:** ^1^ Institut de Biologie Moléculaire des Plantes, CNRS, Université de Strasbourg 12 rue du Général Zimmer F‐67084 Strasbourg France

**Keywords:** tRNA genes, photosynthetic eukaryotes, comparative genomics, tRNA gene dosage, RNA polymerase III, *cis*‐regulatory elements, chromosomal organization, centromeres, tRNA gene clusters

## Abstract

Transfer RNAs (tRNAs) are essential components of the translation machinery. Their abundance and diversity shape decoding capacity as well as the efficiency and accuracy of protein synthesis. Because tRNA abundance is encoded in the genome through tDNA copy number, chromosomal organization, and *cis*‐regulatory sequences controlling transcription, these features are expected to influence the translational system. However, the principles governing nuclear tDNA organization remain poorly understood. Here, we analyzed nuclear tDNA repertoires across 53 photosynthetic eukaryotes spanning major Archaeplastida lineages and secondary endosymbionts, along with seven non‐plant eukaryotic outgroups, using comparative genomic approaches at sequence, chromosomal, and genome‐wide scales. To standardize these analyses and enable interactive exploration of tDNA organization, we developed ShinytRNA (https://nebula.ibmp.unistra.fr/shinytRNA/), a web application for genome‐scale analysis of chromosomal tDNA organization. Nuclear tDNA copy numbers vary by more than two orders of magnitude across species, yet the relative representation of tRNA families corresponding to each amino acid remains strikingly conserved across lineages, revealing strong evolutionary constraints on tDNA dosage. Angiosperm tDNAs exhibit coordinated enrichment of *cis*‐regulatory elements involved in RNA polymerase III transcription, including expanded AT‐rich upstream regions, positional enrichment of CAA motifs, and extended poly(T) termination stretches. At the chromosomal scale, tDNAs are predominantly dispersed along chromosome arms, with homogeneous spacing that scales with genome size, while also showing non‐random chromosomal distribution, exclusion from centromeric regions, and occasional clustering. Together, these patterns reveal conserved yet lineage‐specific principles governing nuclear tDNA organization in plants and highlight how multiple genomic constraints shape the evolution of nuclear tDNA repertoires.

## Significance Statement

How nuclear tRNA genes are organized across plant genomes remains largely unexplored. By comparing tDNA repertoires across 60 photosynthetic and non‐photosynthetic eukaryotes, we show that coordinated reinforcement of RNA polymerase III regulatory elements is unique to angiosperms, challenging the assumption that such patterns are universal, and reveal conserved yet lineage‐specific principles of chromosomal organization. We also provide ShinytRNA, an interactive web application to explore these genomic features across species.

## INTRODUCTION

Transfer RNAs (tRNAs) are essential components of the translational machinery that decode messenger RNA (mRNA) codons into amino acids during protein synthesis. Each tRNA molecule carries a specific amino acid and recognizes one or more synonymous codons through its anticodon, thereby ensuring accurate and efficient translation (Rodnina & Wintermeyer, 2001). The abundance and diversity of cellular tRNA pools are key determinants of translational efficiency and fidelity (Kirchner & Ignatova, 2015; Novoa & Ribas de Pouplana, 2012; Plotkin & Kudla, 2011; Zhou et al., 2013).

Part of the regulation and efficiency of protein translation is encoded in genomes through the composition and organization of nuclear tRNA gene (tDNA) repertoires. Nuclear tDNAs form multi‐copy families whose abundance varies widely across species and lineages (Chan & Lowe, 2016; Goodenbour & Pan, 2006; Santos & Del‐Bem, 2022). Because gene copy number often correlates with relative tRNA abundance, tDNA repertoires are commonly used as a proxy for decoding capacity across tRNA isotypes (i.e., tRNAs carrying the same amino acid) (Duret, 2000; Michaud et al., 2011; Novoa & Ribas de Pouplana, 2012). However, copy number alone provides only a partial view of functional tRNA supply, as individual loci can differ markedly in transcriptional activity, and subsets of tDNAs may be selectively deployed across tissues and developmental programs (Gao et al., 2024; Gerber et al., 2020; Hummel et al., 2020, 2025; Rappol et al., 2024; Reimao‐Pinto et al., 2024; Sagi et al., 2016; Thornlow et al., 2020; Torres et al., 2019).

Transcription of nuclear tDNAs is carried out by RNA polymerase III (Pol III) through a promoter architecture combining conserved internal promoter elements with additional regulatory signals located in the surrounding genomic context (Berg & Brandl, 2021; Kessler & Maraia, 2021). Two internal promoter elements, the A‐ and B‐boxes, recruit transcription factor TFIIIC, which in turn positions TFIIIB upstream to enable Pol III initiation. In addition to these internal elements, upstream and downstream sequences contribute to transcriptional efficiency, including upstream AT‐rich, CAA motifs, and downstream poly(T) termination signals, which collectively modulate transcriptional initiation, termination, and Pol III recycling (Berg & Brandl, 2021; Choisne et al., 1998; Dieci & Sentenac, 1996; Girbig et al., 2022; Giuliodori et al., 2003; Monloy & Planta, 2024; Ulmasov & Folk, 1995; Xie et al., 2022; Yukawa et al., 2000, 2011; Zhang et al., 2011). Recent studies in flowering plants have further characterized these regulatory elements, including CAA motifs, AT‐rich regions, and poly(T) signals (Monloy & Planta, 2024; Zhang et al., 2025).

Beyond local regulatory features, the chromosomal organization of tDNAs may also influence their functional deployment. Nuclear tDNAs can occur as isolated loci or be organized into clusters, sometimes arising through tandem duplication events (Bermudez‐Santana et al., 2010; Hummel & Liu, 2022). Prior work has documented such duplication in angiosperms (Monloy & Planta, 2024; Zhang et al., 2025). In *Arabidopsis thaliana*, clustered tDNAs are epigenetically silenced, illustrating how chromosomal context and chromatin environment can determine which members of the tDNA repertoire remain transcriptionally accessible (Hummel et al., 2020; Hummel & Liu, 2022).

Together, these observations indicate that nuclear tDNA organization involves multiple genomic features, including gene copy number, *cis*‐regulatory sequences associated with Pol III transcription, and large‐scale chromosomal distribution. However, these features have often been examined separately and in a limited number of model species, leaving unclear whether common organizational patterns emerge across plant genomes and photosynthetic lineages. A broader comparative framework is therefore needed to identify conserved and lineage‐specific principles of nuclear tDNA organization from an evolutionary and genomic perspective.

**Figure 1:**
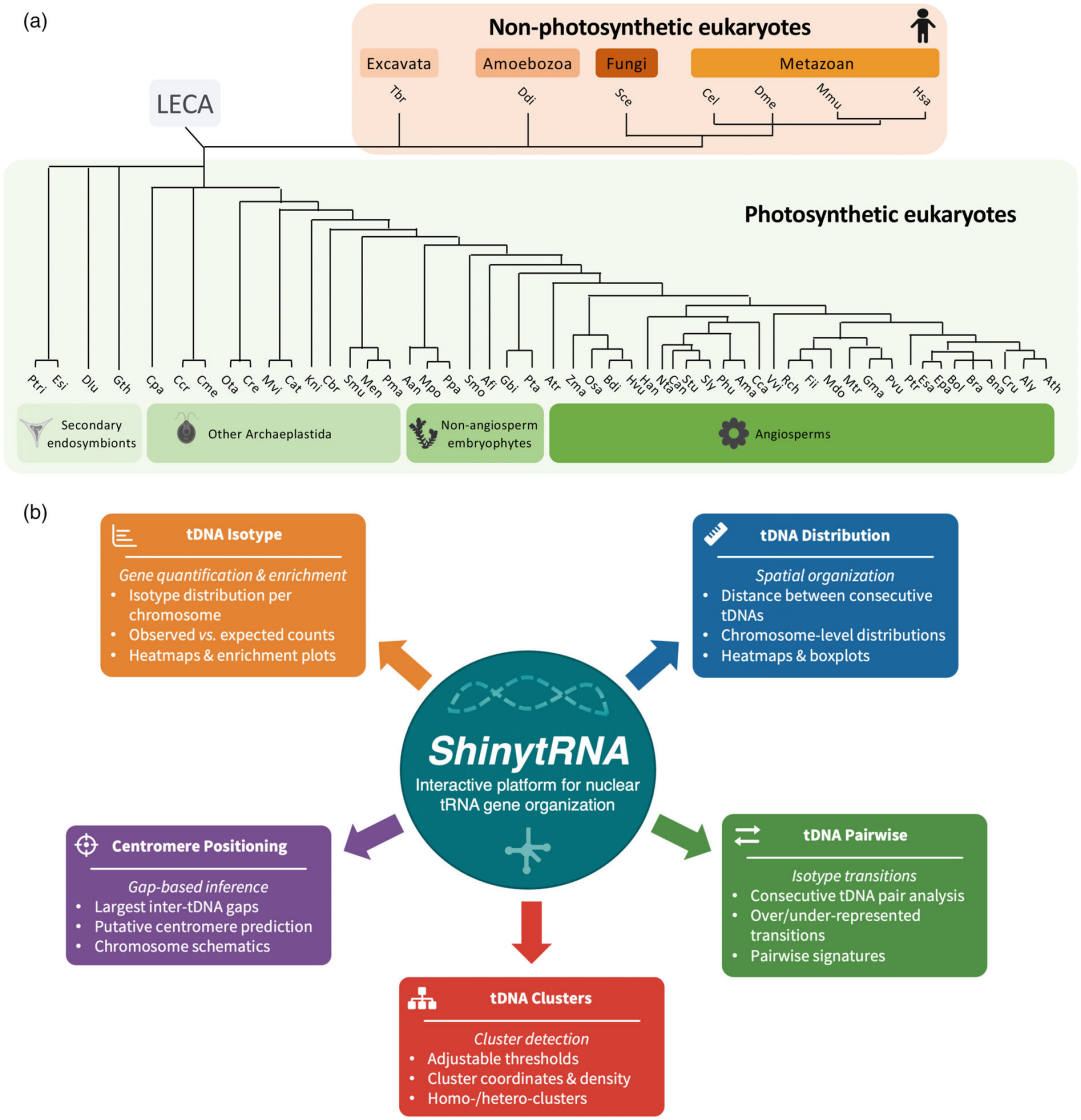
Datasets and analytical frameworks used in this study. (a) Schematic phylogenetic representation of the species included in this study. For photosynthetic organisms, the tree derives from Cognat et al. (2022). Seven non‐photosynthetic eukaryotes were also included for comparison. For several analyses, species were grouped into five major categories: angiosperms, non‐angiosperm embryophytes, other Archaeplastida, photosynthetic eukaryotes with secondary plastids, and non‐photosynthetic eukaryotes. LECA, Last Eukaryotic Common Ancestor. Three‐letter species abbreviations are defined in Table S1. (b) Overview of the ShinytRNA platform (https://nebula.ibmp.unistra.fr/shinytRNA/) developed for comparative analysis of nuclear tDNA organization. ShinytRNA integrates complementary modules enabling genome‐wide exploration of tDNA organization. The tDNA Isotype module quantifies amino acid‐specific distributions and tests enrichment across chromosomes. tDNA Distribution evaluates spatial organization by measuring distances between consecutive tDNA. tDNA Pairwise identifies over‐ and under‐represented transitions between neighboring tDNA. tDNA Clusters detects genomic clusters using adjustable thresholds and reports their composition and density. Centromere Positioning infers putative centromere locations based on large inter‐tDNA gaps and visualizes their distribution along chromosomes.

Here, we address this gap by assembling a curated comparative dataset of nuclear tDNA annotations across a broad phylogenetic sampling of photosynthetic eukaryotes together with representative non‐plant outgroups (Figure 1a). To enable standardized chromosome‐scale exploration of nuclear tDNA organization, we also developed ShinytRNA, a freely accessible interactive web application designed to visualize and export quantitative metrics of tDNA genomic organization (https://nebula.ibmp.unistra.fr/shinytRNA/) (Figure 1b; Figure S1). Developed as an extension of the PlantRNA database (http://plantrna.ibmp.cnrs.fr/) (Cognat et al., 2022), ShinytRNA provides a unified framework for comparative analyses of nuclear tDNA repertoires across genomes. Using the curated comparative dataset and the ShinytRNA framework, we show that nuclear tDNA organization follows conserved yet lineage‐specific principles across photosynthetic eukaryotes. Despite large variation in total tDNA number, the relative abundance of tRNA isotype families remains remarkably conserved, revealing strong constraints on tDNA dosage. Angiosperms display coordinated enrichment of Pol III‐associated *cis*‐regulatory elements, and land plants maintain a predominantly dispersed chromosomal organization characterized by homogeneous intergenic spacing, exclusion from centromeric regions, and limited clustering restricted to specific isotypes. Together, these patterns reveal how multiple genomic constraints collectively shape the evolution and organization of nuclear tDNA repertoires across plant genomes.

## RESULTS

### Conserved hierarchy of tDNA copy numbers among tRNA families across eukaryotes

Nuclear tDNA copy numbers vary by more than two orders of magnitude among eukaryotic species (Chan & Lowe, 2016; Hummel et al., 2019). Whether this variation alters the relative abundance of individual tRNA isotypes or instead preserves a conserved hierarchy across lineages remains unclear. tDNA copy number is known to correlate strongly with measured tRNA abundance in yeast and animals (Behrens et al., 2021; Duret, 2000; Nagai et al., 2021). In photosynthetic organisms, earlier studies reported correlations between tDNA copy numbers, amino acid usage, and codon frequencies, yet these analyses were limited to only four angiosperms (i.e., *Medicago truncatula*, *Populus trichocarpa*, *A. thaliana*, and *Oryza sativa*) and the green alga *Chlamydomonas reinhardtii* (Michaud et al., 2011). Whether such a relationship is conserved across diverse photosynthetic eukaryotic lineages remained unknown. To address this, we quantified nuclear tDNA copy numbers per isotype across representative photosynthetic lineages, using species with curated tDNA annotations spanning aquatic taxa (i.e., glaucophytes, rhodophytes, chlorophytes, streptophyte algae, and secondary endosymbionts) to land plants (i.e., hornworts, liverworts, mosses, lycophytes, ferns, gymnosperms, and angiosperms) (Figure 1a). To avoid biases associated with local tandem amplification, clustered tDNAs were excluded from this specific analysis. The exclusion criterion applied here specifically targets tandem arrays (as defined by the ≥5 genes within 5 kb threshold described below), in order to avoid the over‐representation of particular isotypes driven by local duplication. This choice is further supported by their epigenetic silencing in *A. thaliana* under standard conditions (Hummel et al., 2020). We note, however, the SYY cluster in *A. thaliana* was recently found to be selectively reactivated in specific cell types (Hummel et al., 2025), highlighting that clustered tDNAs may play important roles in tissue‐ or context‐specific expression programs.

**Figure 2:**
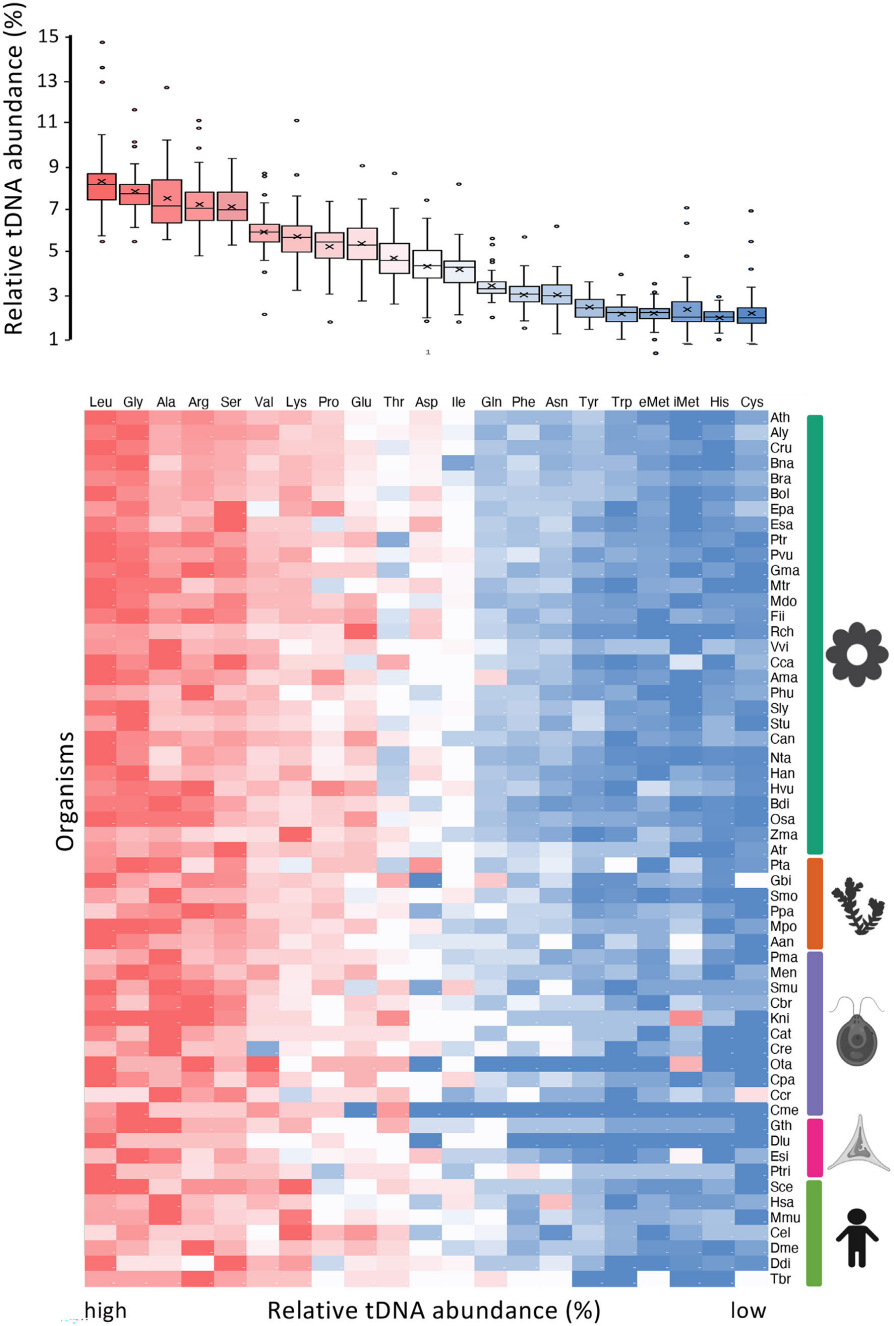
Conserved distribution of tDNA copy‐numbers across eukaryotes. Box plot (top) and heat map (bottom) showing the distribution of nuclear tDNA copy numbers per amino acid across representative photosynthetic organisms and other eukaryotes. Amino acids are ordered according to amino acid frequency in *Arabidopsis thaliana* (Michaud et al., 2011). Elongator methionine (eMet) and initiator methionine (iMet) tRNAs are distinguished. Clustered tDNAs were excluded. Names of photosynthetic organisms are abbreviated as in Table S1.

Despite extreme variation in total tDNA number, ranging from only 43 genes in the highly compact genome of the red alga *Cyanidioschyzon merolae* to approximately 7500 genes in the fern *Azolla filiculoides* (Cognat et al., 2022), the relative ranking of tDNA copy numbers per isotype remains remarkably conserved (tDNA Isotype module of the ShinytRNA platform; Figure 2). Isotypes encoding rare amino acids, such as tryptophan, methionine, histidine, and cysteine, consistently rank among the least abundant, whereas those for leucine, glycine, alanine, arginine, and serine rank among the most abundant. This hierarchy is conserved not only across deeply diverged photosynthetic taxa but also extends to non‐photosynthetic eukaryotic supergroups, including *Excavata*, *Amoebozoa*, and *Opisthokonta* (Figures 1a and 2). Together, these findings reveal a broadly conserved tDNA copy number hierarchy across eukaryotes, pointing to strong and universal translational constraints on tDNA dosage.

### Coordinated enrichment of Pol III *cis*‐regulatory elements in angiosperms

**Figure 3:**
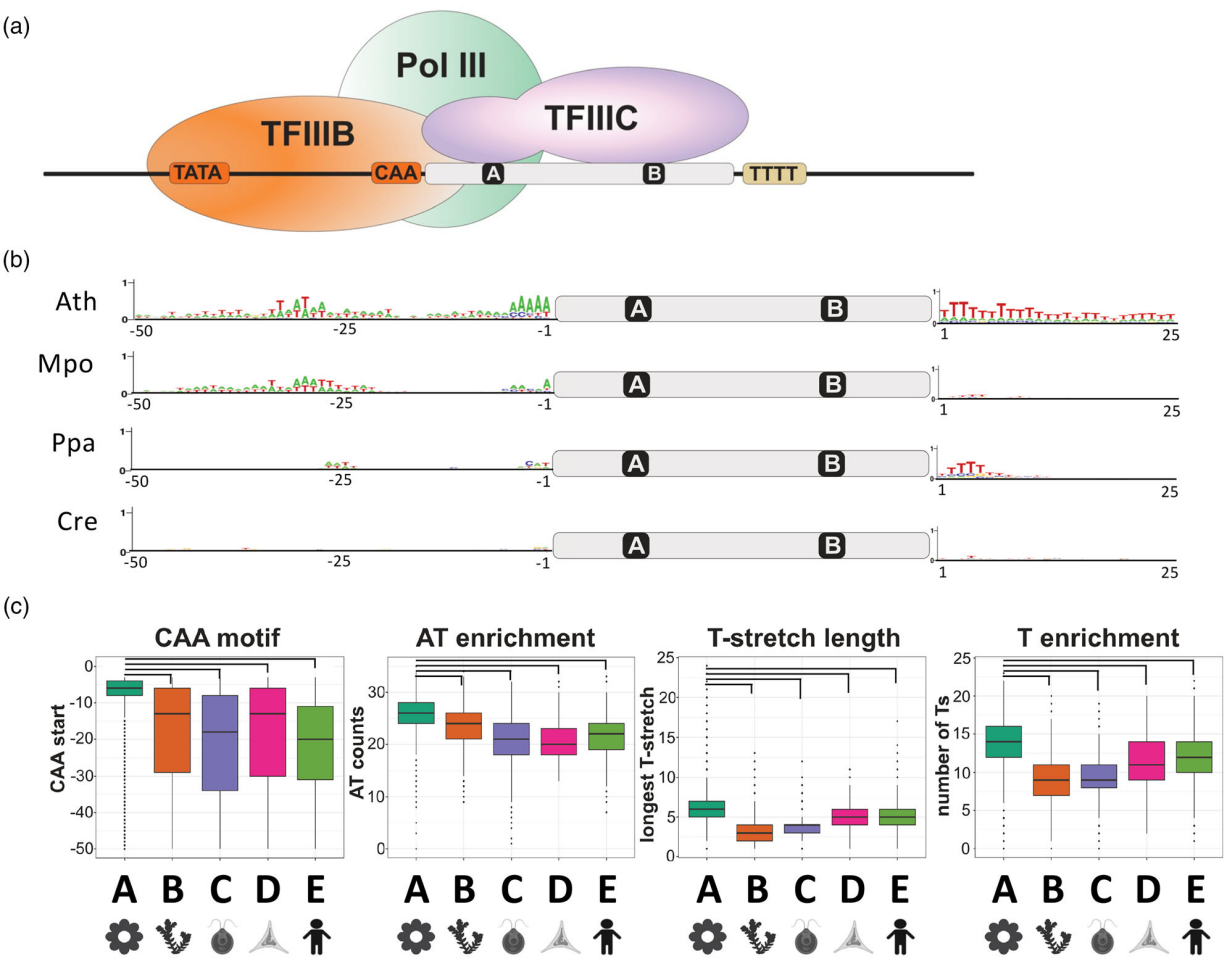
Comparative analysis of *cis*‐regulatory elements associated with tDNA transcription. (a) Schematic representation of the major *cis*‐regulatory elements associated with RNA polymerase III (Pol III) transcription of tDNAs. Upstream regions include A/T‐rich sequences and CAA motifs, while internal A and B boxes and downstream poly‐T stretches define the core Pol III promoter architecture. These elements interact with transcription factors TFIIIB and TFIIIC to ensure transcriptional initiation, elongation, and termination. (b) Sequence logos showing nucleotide conservation in upstream (−50 to −1) and downstream (+1 to +25) flanking regions of tDNAs from *Arabidopsis thaliana* (Ath), *Marchantia polymorpha* (Mpo), *Physcomitrium patens* (Ppa), and *Chlamydomonas reinhardtii* (Cre). The +1 position marks the first nucleotide of the mature tRNA. In (a) and (b), A and B refer to the internal A‐box and B‐box promoter elements, which are located within the tDNA coding sequence and serve as binding sites for the transcription factor TFIIIC. (c) Boxplots comparing *cis*‐regulatory sequence features across five major organismal groups: (A) angiosperms (*n* = 16 367 tDNAs), (B) non‐angiosperm embryophytes (*n* = 8091), (C) other Archaeplastida (*n* = 2018), (D) photosynthetic eukaryotes with secondary plastids (*n* = 411), and (E) non‐photosynthetic eukaryotes (*n* = 1298) (see Figure S2 for details). Parameters include the position of the first upstream CAA motif, upstream A/T content, the length of the longest downstream poly‐T stretch, and downstream T enrichment. Statistical significance relative to angiosperms is indicated by horizontal bars (****P < 0.0001; see Table S2; Figures S2–S6).

Beyond gene dosage, the functional contribution of nuclear tDNAs also depends on the *cis*‐regulatory elements that control their transcription. To investigate whether the *cis*‐regulatory architecture of nuclear tDNAs has diversified across photosynthetic lineages, we analyzed the 5′ upstream (−50 to −1) and 3′ downstream (+1 to +25) flanking regions of nuclear tDNAs (Figure 3). Internal A‐ and B‐box sequences, which are encoded within the tDNA body itself and serve as binding sites for TFIIIC, are depicted schematically in Figure 3a. While these internal elements are highly conserved across all lineages examined (as expected from their role in tRNA secondary structure), we observed no significant lineage‐specific differences in A‐ or B‐box consensus in our dataset. Therefore, our quantitative comparisons focused on the flanking motifs, including upstream CAA motifs, AT‐rich regions, and poly(T) termination signals. Sequences were retrieved from the PlantRNA 2.0 database (Cognat et al., 2022) and analyzed by sequence logos together with quantitative motif profiling across all annotated nuclear tDNA loci.

Upstream *cis*‐regulatory elements display marked lineage‐specific patterns across photosynthetic eukaryotes. Approximately 67% of nuclear tDNAs in angiosperms harbor at least one CAA motif within the −10 to −3 upstream region, compared to substantially lower proportions in other photosynthetic lineages and non‐plant organisms (Figure 3b,c; Figures S2 and S6a,b; Table S2). These findings are consistent with and extend those of Monloy and Planta (2024) and Zhang et al. (2025), who similarly reported enrichment of CAA motifs and AT‐rich upstream regions in angiosperm tDNAs, and confirm these patterns across a broader phylogenetic sampling that includes non‐angiosperm photosynthetic lineages. Notably, CAA motifs are detectable in *Amborella trichopoda*, which is the sister group to the rest of the angiosperm clade (Amborella Genome, 2013), albeit at lower frequency (48%) than in other angiosperms (Figures S2 and S6a,b). In addition to this increased occurrence, the position of the first detected CAA motif is more tightly constrained in angiosperms, indicating enhanced positional specificity. Similarly, upstream AT‐rich regions between positions −35 and −20 show significantly higher enrichment in angiosperms than in non‐angiosperm embryophytes, other Archaeplastida, or lineages derived from secondary endosymbionts (Figure 3b,c; Figure S3; Table S2). In contrast, non‐angiosperm photosynthetic lineages display lower frequencies of CAA motifs and weaker, more variable AT enrichment profiles (Figure 3c). Downstream termination features are similarly enriched in angiosperms. Both the overall thymidine content within the +1 to +25 region and the length of the longest contiguous poly(T) stretch are significantly increased compared to other lineages (Figure 3b,c; Figures S4–S6; Table S2). To further characterize the lineage‐specific distribution of downstream poly(T) elements, we quantified the proportion of tDNAs harboring at least four consecutive T residues downstream (i.e., the minimum generally accepted for functional Pol III termination; Yukawa et al., 2000; Girbig et al., 2022; Xie et al., 2022). This proportion varies substantially across lineages. Angiosperms display the highest frequency, followed by photosynthetic eukaryotes with secondary plastids, other Archaeplastida, and non‐angiosperm embryophytes (Figure S6c,d), consistent with the coordinated strengthening of termination signals observed in angiosperms.

Consistent with prior studies (Monloy & Planta, 2024; Zhang et al., 2025), these results reveal a coordinated strengthening of multiple *cis*‐regulatory elements associated with Pol III transcription in angiosperms. The coincident enhanced conservation of upstream AT‐rich regions, CAA motifs, and poly(T) termination signals is consistent with a refined transcriptional framework for initiation, termination, and reinitiation in flowering plant tDNAs. This may reflect lineage‐specific evolutionary pressures or functional demands rather than a universal hierarchy of transcriptional efficiency. Notably, although these *cis*‐regulatory features are detectable in non‐photosynthetic eukaryotic species, they do not exhibit the same coordinated enrichment observed in angiosperms (Figure 3b,c; Figures S2–S6), indicating that this pattern is not a general property of eukaryotic tDNAs but is instead a distinctive feature of photosynthetic lineages, particularly angiosperms.

### Intergenic spacing of nuclear tDNAs scales with genome size

Like any multi‐copy gene family, the functional output of nuclear tDNAs may also be influenced by their organization, including their spacing and distribution along chromosomes. Whether such chromosome‐scale organization follows conserved principles across plant genomes remains largely unexplored. To investigate chromosome‐scale organization patterns, we restricted our analyses to 31 photosynthetic species with chromosome‐level genome assemblies available in the PlantRNA 2.0 database and analyzed them using the ShinytRNA platform (tDNA Distribution and tDNA Pairwise modules; Figure 1b). This subset spans major plant lineages, including one alga, one bryophyte, one lycophyte, one gymnosperm, and 27 angiosperms. For comparison, six representative non‐plant outgroups were also included, comprising one protist, one fungus, and four metazoans.

**Figure 4:**
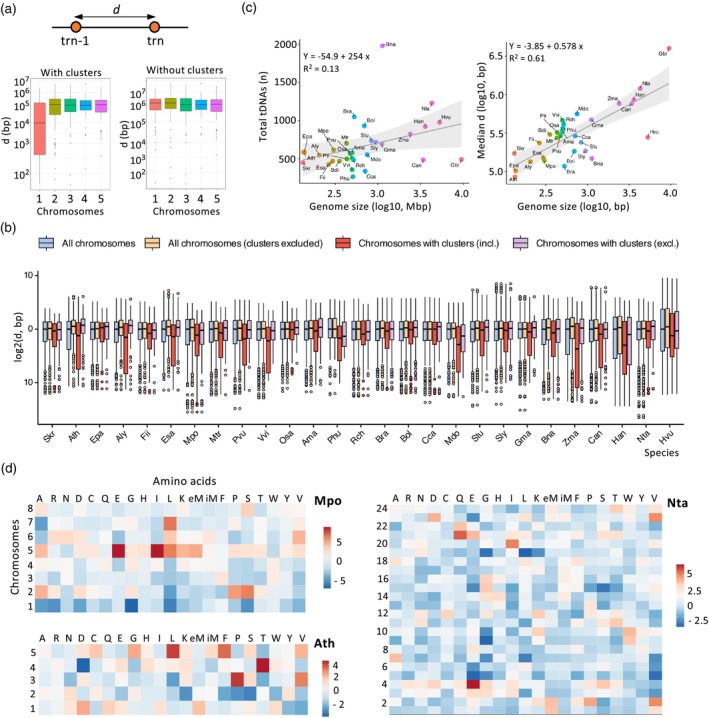
Chromosomal dispersion and spacing of tDNAs across plant genomes. (a) Boxplots showing intergenic distances (*d*) between adjacent tDNAs (trn‐1 to trn) along *Arabidopsis thaliana* chromosomes, calculated either including (left) or excluding (right) tDNA clusters. (b) Variation in intergenic distances between adjacent tDNAs across species. Boxplots compare (i) all chromosomes (blue), (ii) all chromosomes excluding clusters (orange), (iii) only chromosomes containing at least one tDNA cluster, with intra‐cluster distances included (red), and (iv) the same set of chromosomes after excluding intra‐cluster distances (purple). Species are ordered by increasing genome size and species lacking clusters are omitted. (c) Linear regressions (dark gray lines) depicting the relationship between genome size and total tDNA number (left) or median intergenic distance between tDNAs (right). Regression equations and coefficients of determination (*R* ^2^) are indicated. Gray‐shaded areas represent the 95% confidence interval. Each point corresponds to one species. *Chlamydomonas reinhardtii* is excluded here but shown in Figure S7. (d) Heat maps illustrating the chromosomal distribution of isotype families in *Marchantia polymorpha* (Mpo), *A. thaliana* (Ath), and *Nicotiana tabacum* (Nta). Rows represent chromosomes and columns represent amino acid families (single‐letter code). Colors indicate deviation from a random distribution normalized to genome size, with red indicating enrichment and blue depletion. Clustered tDNAs were excluded.

In *A. thaliana*, nuclear tDNAs are distributed across all chromosomes and display relatively uniform inter‐tDNA distances (*d*), defined as the genomic distance between adjacent tDNA loci (Figure 4a). Chromosomes 1 and 2, however, exhibit reduced median *d* values and distributions skewed toward smaller distances due to the presence of previously described tDNA clusters (Hummel et al., 2020; Michaud et al., 2011). When these clustered loci are excluded, median *d* values become comparable across chromosomes, restoring a homogeneous genome‐wide spacing pattern (Figure 4a). Thus, in *A. thaliana*, local clustering increases tDNA density without disrupting overall chromosomal dispersion.

This predominantly homogeneous spacing pattern is conserved across angiosperms and, more broadly, among land plants (Figures S7 and S8). In all examined species, chromosomes harboring tDNA clusters display reduced median *d* values relative to genome‐wide averages, reflecting local compression caused by densely packed loci (Figure 4b, red boxes; Figure S7). For example, this can be observed in *Zea mays*, *Vitis vinifera*, or *Malus domestica* which harbor tDNA^Ala^, tDNA^Ile^, tDNA^Pro^, and tDNA^Ser^ clusters, respectively (Table S3; Figure S7). Exclusion of intra‐cluster intervals reduces variance and restores more homogeneous spacing distributions, indicating that short within‐cluster distances account for most deviations from uniformity (Figure 4b).

To assess how genome expansion relates to tDNA organization, we examined the relationship between genome size, total tDNA number, and median *d* (Figure 4c). In this analysis, ‘total tDNAs’ refer to all annotated nuclear tDNAs in chromosome‐level assemblies, including both clustered and dispersed loci, unless otherwise stated. Across photosynthetic eukaryotes, total tDNA copy number shows no significant correlation with genome size (*R* ^2^ = 0.13), suggesting that increases in genome size are not associated with proportional expansion of tDNA repertoires. In contrast, genome size is positively correlated with median *d* (*R* ^2^ = 0.61), indicating that larger genomes tend to exhibit increased intergenic spacing between tDNA loci. These results suggest that genome expansion is primarily accommodated through increased spacing rather than extensive amplification of tDNA copy number. Notably, the green alga *C. reinhardtii* deviates from this pattern, displaying irregular inter‐tDNA distances and multiple dense clusters (Figures S8 and S9; Cognat et al., 2008). This organization contrasts with the predominantly homogeneous dispersion observed in land plants and more closely resembles the distribution patterns described in metazoan genomes such as human and mouse (Iben & Maraia, 2014; Raab et al., 2012; Van Bortle et al., 2017) (Figure S8).

Collectively, these analyses reveal that land plant genomes exhibit a conserved, predominantly dispersed chromosomal organization of nuclear tDNAs. Although local clustering can generate regional increases in tDNA density, genome expansion is primarily associated with increased spacing between tDNAs rather than their total number.

### Chromosomal partitioning of tDNAs reveals isotype‐specific biases and centromeric exclusion

Beyond intergenic spacing, we next assessed whether tDNA isotype families are randomly distributed across chromosomes relative to genome‐wide expectations. Heat map analyses reveal chromosome‐specific enrichment or depletion of particular isotype families relative to expected counts normalized to chromosome size (Figure 4d; Figure S10; tDNA isotype distribution module of the ShinytRNA platform). In *A. thaliana*, for example, tDNA^Leu^ and tDNA^Phe^ show strong enrichment on chromosome 5, whereas tDNA^Asp^ is relatively depleted from chromosome 4. Such chromosome‐specific biases are not unique to *A. thaliana*; similar patterns of enrichment and depletion are observed in *Marchantia polymorpha* and *Nicotiana tabacum* (Figure 4d), as well as in *O. sativa*, *Selaginella kraussiana*, and *Ginkgo biloba* (Figure S10), indicating that dispersed tDNAs are not uniformly partitioned among chromosomes across diverse plant lineages.

At a broader scale, tDNA loci preferentially localize along chromosome arms and are markedly depleted from centromeric and pericentromeric regions (Figure S11; Centromere positioning module of the ShinytRNA platform). This exclusion pattern is consistently observed despite major differences in centromere size and organization across species, for example in *A. thaliana*, where centromeres are relatively compact and well defined, as well as in *Solanum lycopersicum*, with much larger chromosomes and more extended pericentromeric regions (Figure S11).

Although clustering influences intergenic spacing at a local scale, these chromosome‐level patterns indicate that nuclear tDNAs are also influenced by broader chromosomal architecture. Together, these analyses show that nuclear tDNAs in land plants display non‐random chromosomal partitioning, characterized by isotype‐specific biases and preferential localization outside centromeric domains. This organization suggests that tDNA loci are integrated into large‐scale chromosomal architecture, reflecting conserved spatial constraints that extend beyond simple gene density or local clustering effects.

### tDNA local clustering reveals lineage‐ and family‐specific aggregation patterns

**Figure 5:**
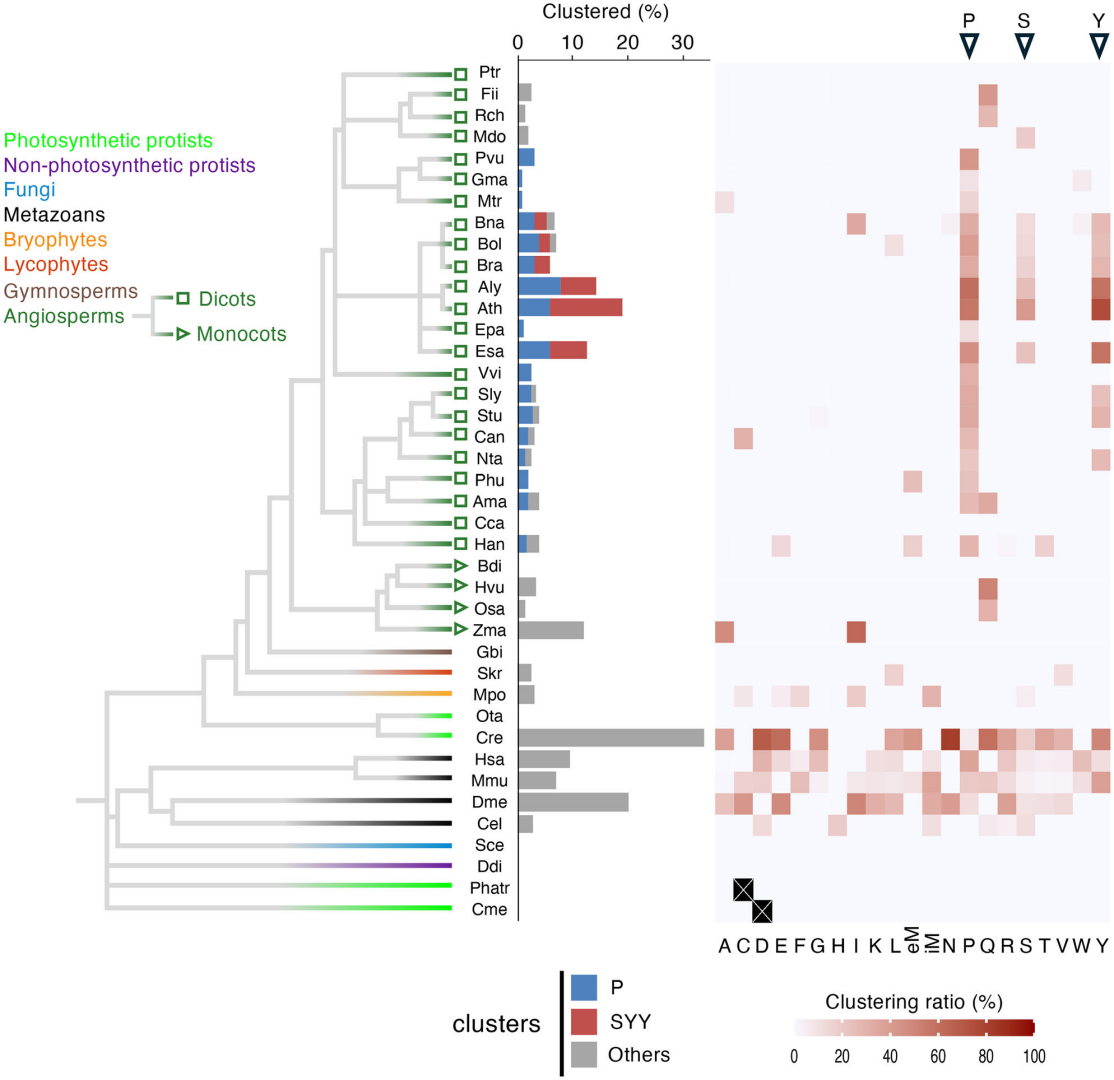
Comparative analysis of tDNA clustering across species and isotype families. Histogram showing the percentage of total clustered tDNAs in each species (left) and heat map showing the clustering ratio of tDNA isotypes in each species (right), under the clustering criterion ≥5 genes within 5 kb. White indicates the absence of clustering (all genes isolated), whereas dark red indicates complete clustering (all genes grouped), with the color scale representing clustering ratio (%) from 0 to 100. White arrows point to three tDNA isotype families (proline/P, serine/S, and tyrosine/Y) that frequently cluster in angiosperms (see blue and red histogram). In *Cyanidioschyzon merolae* (Cme) and *Phaeodactylum tricornutum* (Phatr), asparagine (D) and cysteine (C) tDNAs could not be confidently annotated and were excluded from the analysis (black crossed‐out boxes). Complete results for all clustering criteria are provided in Figure S10.

Although nuclear tDNAs are predominantly dispersed along chromosomes, they also form localized clusters within chromosomal arms. To characterize these clusters, we analyzed their occurrence using the tDNA Clusters module of the ShinytRNA platform across a range of proximity‐based criteria applied to chromosome‐scale genome assemblies. Clusters were defined as groups of ≥3, ≥5, or ≥10 tDNAs located within genomic windows of 1, 5, or 50 kb (Figure 5; Figure S12). Stringent criteria (≥3 genes within 1 kb) identified only a limited number of loci, whereas more permissive thresholds (≥3 genes within 50 kb) generated numerous associations. Based on this systematic evaluation, we retained a threshold of ≥5 genes within 5 kb as a balanced criterion capturing recurrent clusters while limiting spurious associations (Figure 5). Notably, the use of more permissive criteria (≥10 genes within 50 kb) did not substantially increase clustering ratios in gymnosperms and angiosperms, indicating that plant tDNA clusters are typically compact and tightly bounded. By contrast, in several metazoan genomes (e.g., human and mouse) and in non‐plant eukaryotes such as *Saccharomyces cerevisiae* and the protist *Dictyostelium discoideum*, the more permissive criterion (≥10 genes within 50 kb) markedly increased the detectable clustering fraction (Figure S12). This reflects the presence of larger and more spatially extended aggregation domains involving multiple tDNA isotype families, commonly referred to as tDNA islands (Coughlin et al., 2009; Iben & Maraia, 2014), which clearly differ from the compact and isotype‐restricted clusters observed in land plants.

Under the threshold, five genes within 5 kb, the proportion of clustered tDNAs varies markedly across plant species (Figure 5). In most land plants, clustered loci represent a small fraction of total tDNAs, typically below 5%, consistent with a predominantly dispersed genomic organization. In contrast, several *Brassicaceae* species display substantially higher clustering ratios, although this pattern is not universal, as illustrated by *Eutrema parvula*. Outside this lineage, elevated clustering is also observed in *Z. mays*, where clustered tDNAs exceed 10% of total loci. Finally, in the green alga, *C. reinhardtii*, more than 30% of tDNAs are clustered.

To further characterize the composition of tDNA clusters, we calculated for each isotype a clustering ratio, defined as the proportion of genes belonging to clusters relative to the total gene count of that isotype. This analysis confirms that clustering is unevenly distributed across both lineages and isotype families (Figure 5; Figures S12 and S13). In photosynthetic protists, including *Ostreococcus tauri*, *C. merolae*, and *Phaeodactylum tricornutum*, clustering is minimal, with nuclear tDNAs occurring predominantly as singletons. In contrast, land plants display detectable clustering involving a restricted subset of isotypes. Among angiosperms, clustering is dominated by a limited number of recurrent configurations. In particular, tDNA^Pro^ frequently forms homoclusters composed of adjacent copies of the same isotype, whereas tDNA^Ser^ and tDNA^Tyr^ commonly co‐occur in structured heteroclusters (Figure S13; Table S3). Additional isotypes, such as tDNA^Gln^, also show clustering in specific lineages (e.g., in *Hordeum vulgare* and *O. sativa* or in *Fragaria iinumae* and *Rosa chinensis*), while other clusters remain species‐specific, as illustrated by tDNA^Ala^ clusters in *Z. mays* or tDNA^Ile^ clusters in *Z. mays* and *Brassica napus* (Figure 5; Table S3).

As previously reported (Monloy & Planta, 2024; Zhang et al., 2025), our results show that tDNA clustering in plants is structured and selective, involving a limited set of isotypes organized into compact and recurrent configurations. In contrast, several metazoan genomes and the green alga *C. reinhardtii* exhibit broader aggregation domains under relaxed proximity thresholds, reflecting more spatially extended and heterogeneous tDNA assemblies (Figures S12 and S13). These lineage‐specific differences reveal distinct modes of local tDNA organization superimposed on a globally dispersed chromosomal architecture.

### Clustered and dispersed tDNAs exhibit distinct *cis*‐regulatory conservation patterns

**Figure 6:**
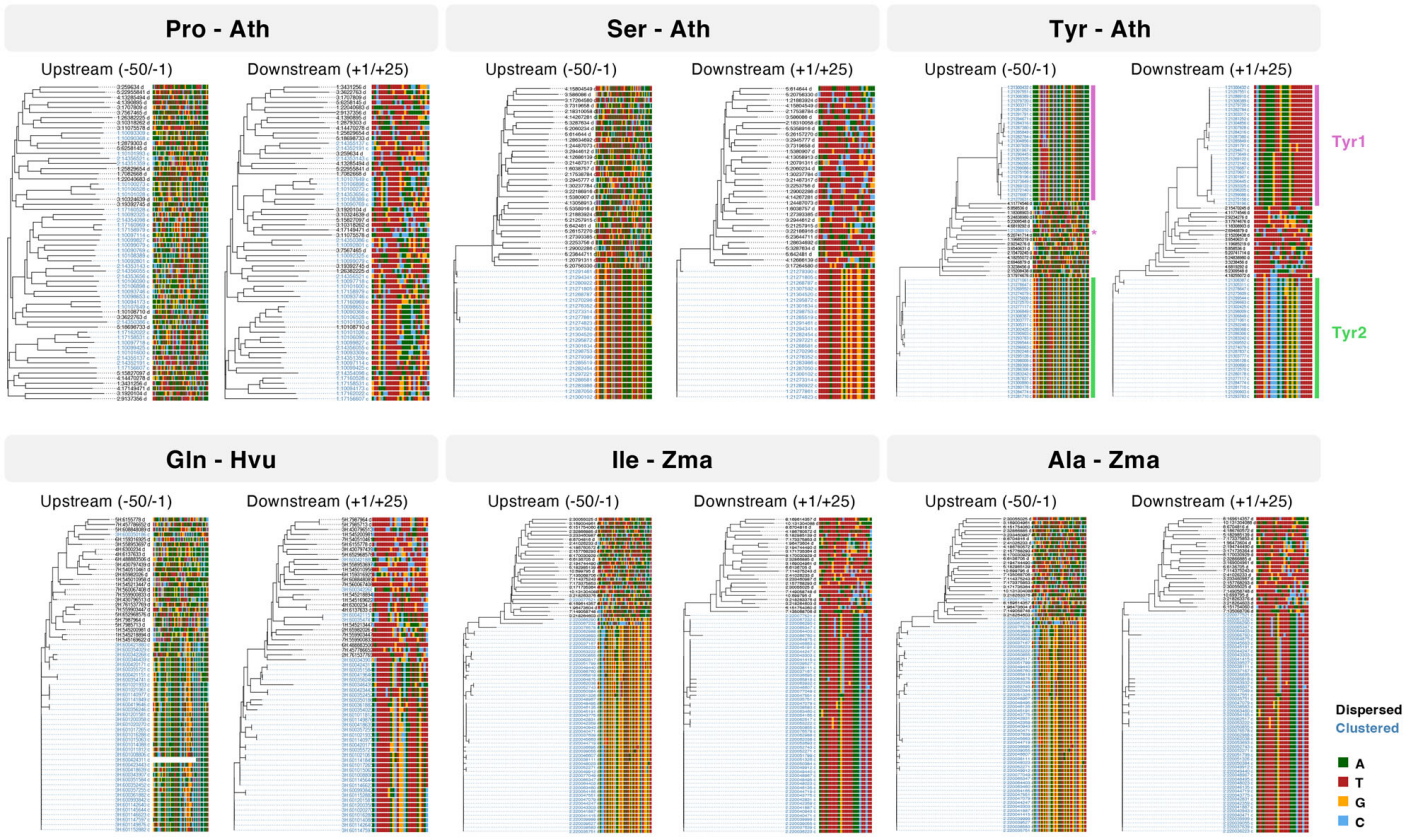
Clustered tDNAs exhibit increased cis‐regulatory sequence conservation relative to dispersed loci. Phylogenetic trees generated from flanking sequences of nuclear tDNAs, with corresponding sequence logos illustrating nucleotide conservation in these regions. Loci belonging to clusters are shown in blue, while dispersed loci are in black. Trees were constructed separately from upstream (−50 to −1) and downstream (+1 to +25) flanking sequences, with sequence logos summarizing nucleotide frequencies in the same regions. Representative clusters include tDNA^Pro^ in *Arabidopsis thaliana* (Ath), tDNA^Gln^ in *Hordeum vulgare* (Hvu), and tDNA^Ile^ in *Zea mays* (Zma), organized as tandem arrays of the same isotype, as well as the Ser‐Tyr‐Tyr cluster in *A. thaliana*, corresponding to tandem arrays of a repeated Ser‐Tyr‐Tyr module. Within the Ser‐Tyr‐Tyr cluster of *A. thaliana*, the first Tyr copy (Tyr1) groups toward the top of the phylogenetic tree (indicated in violet), whereas the second Tyr copy (Tyr2) groups toward the bottom (indicated in green). The violet asterisk (*) indicates the single Tyr 1 copy that does not group with the other Tyr 1 copies.

The identification of lineage‐ and isotype‐specific tDNA clusters prompted us to examine whether clustering is associated with distinct *cis*‐regulatory sequence patterns beyond simple local duplication. We compared the upstream (−50 bp) and downstream (+25 bp) flanking regions of clustered and dispersed tDNAs within individual isotypes (Figure 6). In *A. thaliana*, clustered tDNAs^Pro^ form tandem arrays in which downstream flanking regions are markedly more homogeneous than those of dispersed tDNAs^Pro^, particularly with respect to poly(T) tract organization. By contrast, upstream regions display weaker conservation and greater sequence variability. This asymmetry suggests that selective constraints associated with transcription termination and Pol III recycling may be stronger than those affecting transcription initiation within these clusters. The Ser‐Tyr‐Tyr (SYY) cluster provides a distinct example of structured conservation. Its organization consists of repeated blocks of an entire SYY unit (Hummel et al., 2020). When tDNAs^Tyr^ are analyzed according to their position within the repeated block, the first and second Tyr copies form distinct, monophyletic clades in both upstream and downstream trees (Figure 6; first (Tyr1) and second (Tyr2) Tyr copies are indicated in violet and green, respectively). This positional specificity indicates that copies at the same position across different repeat units are more similar to each other than Tyr1 and Tyr2 copies within the same unit. Both upstream and downstream flanking regions show strong conservation, supporting a model of segmental duplication of entire SYY units, followed by selective maintenance of the complete *cis*‐regulatory module rather than independent aggregation of individual loci. Similar patterns are observed in monocots. In *Z. mays*, large tandem arrays of tDNAs^Ile^ and tDNAs^Ala^ display highly uniform upstream and downstream sequences compared to dispersed copies. In *H. vulgare*, clustered tDNAs^Gln^ likewise show greater *cis*‐regulatory similarity within clusters than among dispersed loci.

Together, these observations indicate that tandem duplication likely contributes to cluster formation but does not fully explain the recurrent conservation of cluster composition and *cis*‐regulatory architecture. The recurrence of some cluster configurations, notably Pro‐containing clusters and SYY‐like arrangements, across distantly related angiosperms (Figure 5) suggests that these clusters are unlikely to result solely from recent species‐specific duplication events. Instead, they may reflect either recurrent emergence under similar selective constraints or older arrangements maintained across lineages. This broader evolutionary persistence is consistent with the fact that different cluster types display distinct patterns of *cis*‐regulatory conservation, likely reflecting different functional constraints. For example, clusters such as SYY maintain strong conservation of both upstream and downstream regions, suggesting selective preservation of complete transcriptional units, whereas clusters such as Pro show stronger conservation of downstream termination signals, consistent with stronger constraints on transcription termination and Pol III recycling. These results suggest that clustered tDNAs follow evolutionary trajectories distinct from dispersed loci, shaped by both lineage‐specific functional constraints and long‐term maintenance of specific regulatory architectures.

## DISCUSSION

Despite their essential role in translation and emerging functions in genome organization and chromatin regulation (Guimaraes et al., 2021; Hamdani et al., 2019; Iwasaki et al., 2020; Raab et al., 2012; Shukla & Bhargava, 2018), the principles governing nuclear tDNA organization across photosynthetic eukaryotes remain poorly understood. In particular, it is unclear whether tDNA repertoires primarily reflect neutral genomic processes or are shaped by selective constraints linked to translational demand and chromatin context. Here, by systematically analyzing tDNA repertoires across diverse photosynthetic lineages using the ShinytRNA platform, we uncover multiple layers of non‐random organization operating across genomic scales.

In spite of variations in total nuclear tDNA copy numbers, we found that their relative ranking per isotype remains remarkably conserved across photosynthetic eukaryotes (Figure 2). This hierarchy extends to diverse non‐photosynthetic eukaryotic supergroups, including *Excavata*, *Amoebozoa*, and *Opisthokonta* (Du et al., 2017), indicating that it represents a conserved and functionally constrained organization of decoding capacity. Consistent with this, high‐copy isotypes correspond to frequently used amino acids, whereas low‐copy isotypes for rare amino acids (tryptophan, methionine, histidine, cysteine) may enable regulatory ribosome pausing and co‐translational protein folding (Plotkin & Kudla, 2011; Zhou et al., 2013). Interestingly, this hierarchy shows substantial agreement with Trifonov's consensus chronology of amino acid appearance in the genetic code (Trifonov, 2000), with early‐recruited amino acids (glycine, alanine, proline, serine, valine, leucine, glutamic acid) tending to have high tDNA copy numbers. Comparison with the more recent LUCA‐based reconstruction (Wehbi et al., 2024) reveals some discrepancies, notably high tDNA abundance for leucine and serine classified as late additions, and low abundance for methionine, cysteine, and histidine classified as early. Nevertheless, the overall pattern supports the idea that tDNA copy numbers may still retain a detectable imprint of primordial proteome composition and early translational constraints. Subsequent lineage‐specific evolutionary pressures and translational demands have only moderately reshaped this hierarchy. Together, these observations support a model in which codon usage, amino acid composition, and tDNA dosage remain tightly interconnected.

Despite the strong conservation of the tDNA copy number hierarchy across eukaryotes, total tDNA numbers scale weakly or non‐significantly with genome size in photosynthetic lineages (Figure 4) (Michaud et al., 2011; Monloy & Planta, 2024). This weak relationship contrasts with the positive correlation reported in many animals and fungi (Bermudez‐Santana et al., 2010; Goodenbour & Pan, 2006; Santos & Del‐Bem, 2022) and indicates that tDNA repertoires in photosynthetic eukaryotes are not primarily driven by genome expansion, but by multiple interacting evolutionary processes. Transposable element (TE) proliferation, the main driver of genome expansion in plants, can increase intergenic space without proportional amplification of tDNA copy number (Lee & Kim, 2014), while local tandem duplication may promote selective expansion of specific isotypes (e.g., the Pro cluster discussed below; Zhang et al., 2025). In addition, whole‐genome duplication, followed by biased gene retention or loss during diploidization, may further prevent linear scaling between tDNA copy number and genome size (Panchy et al., 2016). Together, these mechanisms may contribute to the distinct relationship between genome size and tDNA repertoire observed in photosynthetic lineages, where genome expansion appears to occur primarily through increased intergenic space rather than proportional amplification of tDNA copy number. In this context, modulation of transcriptional activity through cis‐regulatory elements may represent an additional layer of regulation ensuring sufficient tRNA output despite relatively constrained tDNA repertoire expansion (Monloy & Planta, 2024; Santos & Del‐Bem, 2022).

Consistent with this, our analyses reveal a pronounced enrichment of Pol III‐associated *cis*‐regulatory elements in angiosperms, whereas other photosynthetic lineages display weaker patterns resembling those of non‐plant eukaryotes (Figure 3c). As previously reported (Monloy & Planta, 2024; Zhang et al., 2025), these findings include enrichment of CAA motifs, AT‐rich regions, and poly(T) signals in flowering plant tDNAs. However, by including non‐angiosperm photosynthetic lineages and non‐plant outgroups, we demonstrate that this coordinated enrichment is specific to angiosperms and not a universal feature of photosynthetic or eukaryotic tDNAs. This selective strengthening of transcriptional efficiency appears to have emerged progressively during angiosperm evolution. *Amborella trichopoda*, the sister group to the rest of the angiosperm clade, already exhibits detectable, albeit weaker, enrichment of CAA motifs, indicating that some of this change had already occurred prior to the most recent common ancestor of angiosperms but then intensified in early angiosperm evolution after divergence from *A. trichopoda*. Functionally, these elements are known to modulate multiple steps of Pol III transcription, including initiation, termination, and polymerase recycling (Yukawa et al., 2000). Their coordinated enrichment therefore may reflect increased reliance on these defined sequence motifs for efficient Pol III transcriptional activity in angiosperms. Whether this represents an optimized regulatory architecture or instead reflects a lineage‐specific shift in the mechanisms by which tRNA gene regulation is achieved remains an open question. It may also signify increased constraints on transcriptional efficiency and regulatory precision associated with genome expansion and organismal complexity in angiosperms (Bowman et al., 2017; Dieci et al., 2013; Turowski et al., 2016; Xue et al., 2026). In particular, the strengthening of both initiation‐ and termination‐associated elements may enhance transcriptional robustness while limiting interference with neighboring genes, especially those transcribed by Pol II (Turowski et al., 2016). Beyond transcriptional efficiency, this coordinated enrichment and more consistent positioning may help maintain tDNA output amid genome expansion. Given the weak scaling between tDNA copy number and genome size in plants (Figure 4), modulation of *cis*‐regulatory strength may serve as an important layer to ensure effective tRNA supply without requiring changes in gene copy number (Hummel et al., 2019; Monloy & Planta, 2024). Together, these observations support a model in which tDNA evolution in angiosperms rely on a dual regulatory strategy combining relatively stable gene dosage with increased strength of *cis*‐regulatory elements.

At a more local scale, we observed reduced *cis*‐regulatory divergence within clustered tDNAs compared to dispersed ones (Figure 6). However, the nature of this conservation varies among cluster types. In *A. thaliana*, the SYY cluster displays strong conservation of both upstream and downstream regions, preserving the internal organization of the repeated unit. This conservation occurs despite the heterochromatic context of this region (Hummel et al., 2020) and the elevated mutation rates typically associated with closed chromatin (Makova & Hardison, 2015). Notably, the SYY cluster is selectively reactivated in root cap columella cells, where it has been proposed to supply tRNA^Ser^ and tRNA^Tyr^ for the translation of Ser/Tyr‐rich proteins of the hydroxyproline‐rich glycoprotein (HRGP) family known to be released by these cells in the mucilage (Hummel et al., 2025). This specialized, cell‐type‐specific role may subject the SYY cluster to strong purifying selection, potentially counteracting the mutagenic effects of heterochromatin. The near *Brassicaceae*‐specific distribution of this cluster further raises the possibility of a lineage‐enriched functional specialization, potentially linked to the distinctive organization and secretory activity of the root cap in this family (Kumar & Iyer‐Pascuzzi, 2020; Kumpf & Nowack, 2015). Although this hypothesis remains highly speculative, such developmental constraints could contribute to the selective maintenance of the SYY cluster in this lineage. By contrast, the Pro cluster exhibits weaker conservation of upstream regions but more homogeneous downstream elements, pointing to stronger constraints on transcription termination. This pattern is consistent with its localization within intragenic euchromatic regions (Hummel et al., 2020) associated with Pol II transcription, where chromatin accessibility and transcriptional interference may impose distinct regulatory constraints. Efficient termination in this context is likely critical to promote Pol III recycling and re‐initiation while preventing read‐through transcription and interference within tandem arrays (Dieci et al., 2013; Turowski & Tollervey, 2016; Yukawa et al., 2000). From a functional perspective, the conservation of Pro cluster architecture across dicot angiosperms (Figures 5 and 6) may reflect conditional translational demand for proline‐rich proteins. Although largely silent under normal conditions in *A. thaliana* (Hummel et al., 2020), this cluster may contribute to rapid synthesis of proline‐rich proteins, such as extensins and HRGPs, which are involved in cell wall reinforcement under stress conditions (Hummel et al., 2025; Johnson et al., 2017; Showalter et al., 2010). More broadly, tDNA clustering appears to be the exception rather than the rule across photosynthetic eukaryotes and concerns only a limited subset of isotypes (Figure 5). This aligns with prior observations of isotype‐specific clustering in angiosperms (Monloy & Planta, 2024; Zhang et al., 2025). The recurrence of similar cluster configurations across distantly related species further points to shared selective constraints that may favor the emergence and maintenance of these arrangements. Together, these observations suggest that tDNA clustering is neither random nor universal but instead reflects specific functional constraints and lineage‐specific genomic dynamics.

Consistent with the limited extent of tDNA clustering, our analyses reveal that land plant genomes exhibit a predominantly dispersed organization of nuclear tDNAs, characterized by relatively regular inter‐tDNA distances (Figure 4). Moreover, rather than scaling tDNA copy number with genome size, land plants primarily accommodate genome expansion by increasing the spacing between tDNA loci. This pattern contrasts with that observed in many animals and fungi, where tDNA copy number often scales positively with genome size, and inter‐tDNA distances appear more heterogeneous (Bermudez‐Santana et al., 2010; Goodenbour & Pan, 2006; Santos & Del‐Bem, 2022). Interestingly, a similarly irregular distribution of tDNA is also observed in the green alga *C. reinhardtii* (Figure S8), suggesting that the predominantly dispersed organization may represent a specific feature of land plant genomes rather than a general property of photosynthetic eukaryotes. The evolutionary forces shaping the distinct tDNA organization observed in land plants remain unclear but may partly reflect selective constraints linked to translational efficiency. Once an optimal tRNA supply matching codon demand is reached, further increases in tDNA copy number are likely disfavored, as they impose unnecessary energetic costs and increase the risk of translational imbalance (Du et al., 2017; Goodenbour & Pan, 2006). In sessile organisms such as land plants, where growth and stress responses depend on tight resource allocation under fluctuating environmental conditions, limiting unnecessary transcriptional and energetic costs may represent an additional selective advantage.

Taken together, our results suggest that nuclear tDNA organization in photosynthetic eukaryotes emerges from the interplay of multiple constraints operating across genomic scales. At the molecular level, selective pressures on translation shape tDNA repertoire composition and *cis*‐regulatory elements controlling Pol III transcription. At the chromosomal level, genome architecture might govern spatial distribution, ensuring compatibility with chromatin environments and large‐scale genome organization. These constraints are further modulated by lineage‐specific evolutionary processes, including duplication, genome expansion, and ecological adaptation, which introduce additional variability in tDNA copy number and clustering patterns. This integrated framework reveals tDNAs as dynamic genomic elements positioned at the interface between translational demand, chromatin organization, and genome evolution. Rather than evolving independently, tDNA copy number, regulatory architecture, and spatial organization appear to be jointly optimized, defining a structured evolutionary landscape that underlies the diversification and robustness of translational systems across photosynthetic lineages.

## EXPERIMENTAL PROCEDURES

### Data sources

Nuclear tDNA annotations for photosynthetic eukaryotes were retrieved from the PlantRNA 2.0 database (http://plantrna.ibmp.cnrs.fr) (Cognat et al., 2022), which provides curated tDNA annotations together with associated genomic features, including flanking sequences. Annotations in PlantRNA 2.0 are curated to retain only high‐confidence tDNA loci; entries flagged as pseudogenes or tDNA‐like sequences with low structural confidence scores by tRNAscan‐SE (Chan & Lowe, 2019) were excluded during the database curation step (Cognat et al., 2022). For tDNA predictions performed directly from genome assemblies in this study (for *D. discoideum* and *Trypanosoma brucei*), only loci classified as high‐confidence tRNA genes by tRNAscan‐SE (infernal score >20, no pseudogene flag) were retained. The dataset comprises 49 representative species spanning the major Archaeplastida lineages (glaucophytes, rhodophytes, chlorophytes, streptophyte algae, bryophytes, lycophytes, gymnosperms, and angiosperms) as well as four lineages derived from secondary endosymbiosis (Table S1). To provide phylogenetic outgroups, tDNA annotations for five non‐plant organisms were retrieved from the Genomic tRNA Database (http://gtrnadb.ucsc.edu) (Chan & Lowe, 2016), including *S. cerevisiae* (S288c), *Drosophila melanogaster* (dm6), *Caenorhabditis elegans* (ce11), *Mus musculus* (mm39), and *Homo sapiens* (hg38). In addition, two other non‐plant eukaryotic lineages were included: the amoebozoan *D. discoideum* and the excavate *T. brucei*. For these species, nuclear tDNAs were predicted from genome assemblies (GCA_000004695.1 and GCF_000002445.2, respectively) using tRNAscan‐SE v2.0 (Chan & Lowe, 2019) with default eukaryotic parameters. Annotation procedures followed those described in Cognat et al. (2022). The final dataset comprised 60 species and a total of 38 233 nuclear tDNAs.

### Computational meta‐analysis of tDNA organization

Custom R scripts were developed to process and integrate tDNA annotations from PlantRNA datasets across species. Only nuclear‐encoded tDNAs were retained. All entries corresponding to mitochondrial or plastidial tDNAs were excluded. Scaffold sequences, or unplaced contigs, were excluded from genome organization analysis. All analyses were performed in R (v4.5.0). Data processing and genomic interval analyses relied on the packages *data.table* (v1.17.8), *GenomicRanges*, and *rtracklayer*, and graphical outputs were generated using *ggplot2* (v3.5.2), *ggseqlogo*, and *ggtree*. For each species, genomic coordinates of nuclear tDNAs were converted into strand‐aware GRanges objects and ordered along chromosomes. Inter‐tDNA distance (*d*) was defined as the genomic distance between consecutive tDNAs along each chromosome, computed using rank‐ordered coordinates. Distances were calculated genome‐wide and per chromosome, and log10‐transformed for visualization. Chromosome‐level distributions of tDNA isotypes were assessed by comparing observed counts per chromosome to expected counts based on chromosome length. Expected values were calculated assuming a uniform distribution proportional to chromosome size. Deviations from expectation were quantified using standardized residuals derived from chi‐square statistics. Heat map values correspond to standardized Pearson residuals comparing observed and expected chromosome‐level isotype counts. Deviations were considered significant when exceeding the chi‐square threshold for 1 degree of freedom (*χ* ^2^ >3.84; corresponding to |standardized Pearson residual| >1.96), allowing classification of chromosome‐specific enrichment or depletion. Pairwise adjacency relationships between consecutive tDNAs were also computed genome‐wide to characterize local neighborhood organization. Detailed statistical procedures for adjacency analyses are provided in the Supporting Information—Supplementary Methods. To characterize local genomic organization, tDNA clusters were defined using proximity‐based criteria. Unless otherwise stated, clusters were defined as groups of at least five tDNAs within ≤5 kb, a threshold selected as a robust compromise between sensitivity and specificity for detecting compact clusters across species. Alternative thresholds (≥3 genes within 1 kb; ≥10 genes within 50 kb) were also evaluated to assess the robustness of clustering patterns (Figure S12). For each species, clustering ratios were calculated as the proportion of tDNAs assigned to clusters relative to the total number of annotated nuclear tDNAs. Detailed formulas and implementation procedures are provided in Supporting Information—Supplementary Methods.

### Extraction of flanking sequences and *cis*‐regulatory feature analyses

To investigate *cis*‐regulatory features associated with Pol III transcription, genomic flanking regions were extracted for each annotated nuclear tDNA using strand‐aware coordinates. For each gene, 50 nucleotides upstream (−50 to −1 relative to the 5′ end of the tDNA coding sequence) and 25 nucleotides downstream (+1 to +25 relative to the 3′ end) were retrieved from the corresponding genome assembly. Upstream and downstream sequences were analyzed separately to quantify features associated with transcriptional initiation and termination. Sequence logos were generated from aligned flanking regions using the *ggseqlogo* package. Three *cis*‐regulatory features were quantified for each locus: (i) CAA motif occurrence and positional bias, assessed by recording both the presence or absence of at least one CAA trinucleotide within the −50 to −10 upstream region and, when present, the position of the first detected motif; (ii) upstream AT enrichment, calculated as the proportion of adenine and thymine nucleotides; and (iii) downstream termination features, including total thymidine content within the +1 to +25 region and the length of the longest contiguous poly(T) stretch. For comparative analyses, species were grouped into five major phylogenetic categories: angiosperms, non‐angiosperm embryophytes, other Archaeplastida, photosynthetic eukaryotes with secondary plastids, and non‐photosynthetic eukaryotes (Figure 1a; Table S1). Group‐wise comparisons of *cis*‐regulatory features were performed using Kruskal–Wallis tests followed by pairwise Wilcoxon rank‐sum tests with Benjamini–Hochberg correction for multiple testing. Complete statistical results are provided in Table S2. To assess whether *cis*‐regulatory patterns differed according to genomic context, clustered and dispersed tDNAs were analyzed separately for representative isotype families. Flanking sequence similarity was quantified using uncorrected p‐distances calculated with the *dist.dna* function from the *ape* package (model = ‘N’). Distance trees were inferred using the BIONJ algorithm and visualized with *ggtree*.

### ShinytRNA web application

ShinytRNA was developed as an interactive web application in R using the shiny framework (v1.11.1) to facilitate standardized exploration of nuclear tDNA genomic organization across species. The application was implemented using *data.table* (v1.17.8), *ggplot2* (v3.5.2), and *DT* (v0.33) for interactive visualization and export of tabular outputs. ShinytRNA integrates five complementary modules enabling genome‐scale analysis of tDNA organization: (i) a tDNA isotype distribution module for chromosome‐level enrichment analyses, (ii) a tDNA distribution module for spatial dispersion analyses, (iii) a pairwise (adjacency) module for local neighborhood analyses, (iv) a tDNA clusters module for proximity‐based cluster detection, and (v) a centromere positioning module, based on experimentally validated centromere coordinates when available or proxy inference from large tDNA‐free genomic intervals otherwise (Figure 1b; Figure S1). The application allows dynamic parameter adjustment and export of publication‐ready graphical and tabulated outputs.

## AUTHOR CONTRIBUTIONS

GH designed work, performed bioinformatic analyses, analyzed the data, and participated in writing the manuscript. DP developed the ShinytRNA platform, performed bioinformatics analyses, and participated in writing the manuscript. VC participated in tDNA annotation and processing of genomes. LD and AB supervised the work, designed experiments, analyzed the data, and wrote the manuscript.

## CONFLICT OF INTEREST

The authors reported no potential conflict of interest.

## Supporting information


**Data S1.** Supplemental methods.

**Figure S1.** Examples of data analysis performed on the ShinytRNA platform for *Arabidopsis thaliana* (Ath) (https://nebula.ibmp.unistra.fr/shinytRNA/).
**Figure S2.** Boxplot showing the CAA start position of the first CAA motif in the region upstream of the 5′ end of tDNAs.
**Figure S3.** Boxplot showing the AT content in the region from −50 to −35 bp upstream of the 5′ end of tDNAs.
**Figure S4.** Boxplot showing the T content within 25 bp downstream of the 3′ end of tDNAs.
**Figure S5.** Boxplot showing the length of the longest T stretch located within 25 bp downstream of the 3′ end of tDNAs.
**Figure S6.** Histograms showing the proportion of tDNAs with a CAA motif within the −10 to −3 upstream region in photosynthetic species (a) and in major photosynthetic groups (b), and the proportion of tDNAs harboring at least one poly(T) stretch of ≥4 consecutive T residues within the +1 to +25 downstream region in photosynthetic species (c) and in major photosynthetic groups (d).
**Figure S7.** Boxplots showing the distance (*d*) between consecutive tDNAs on chromosomes from land plant species.
**Figure S8.** Comparison of tDNA organization in *Chlamydomonas reinhardtii* versus a representative plant species (*Nicotiana tabacum*) and two non‐plant species (*Mus musculus* and *Homo sapiens*).
**Figure S9.** tDNA organization in the green lineage including *Chlamydomonas reinhardtii*.
**Figure S10.** Heat maps showing the chromosomal distribution of tDNA isotypes in three species (*Oryza sativa*, *Selaginella kraussiana*, and *Ginkgo biloba*).
**Figure S11.** Chromosomal distribution of tDNAs reveals large‐scale genomic organization in plants.
**Figure S12.** tDNA cluster identification upon different filtering conditions.
**Figure S13.** Identification of proline‐, serine‐, and tyrosine‐containing tDNA clusters using ShinytRNA.

**Table S1.** Photosynthetic and non‐photosynthetic species analyzed in this study.

**Table S2.** All pairwise Wilcoxon rank sum tests with Benjamini & Hochberg (BH) adjustment.

**Table S3.** Detailed annotation of tRNA gene clusters identified under different filtering conditions.

## Data Availability

ShinytRNA is freely accessible at https://nebula.ibmp.unistra.fr/shinytRNA/. All scripts and processed datasets are available at https://github.com/ibmp/tDNA-organization-meta-analysis.git to ensure full reproducibility. All the relevant data can be found within the manuscript and its Supporting Information.
