# Supplementary material for: Multilevel genomic constraints shape nuclear tRNA gene organization in plants
Source: Plant J. 2026 Aug 4;127(3):e71075. doi: 10.1111/tpj.71075 (PMC13437025; doi:10.1111/tpj.71075)
Supplement: Supplementary file 1 [file TPJ-127-0-s001.pdf]

tDNA Isotype

Isotype distribution per chromosome

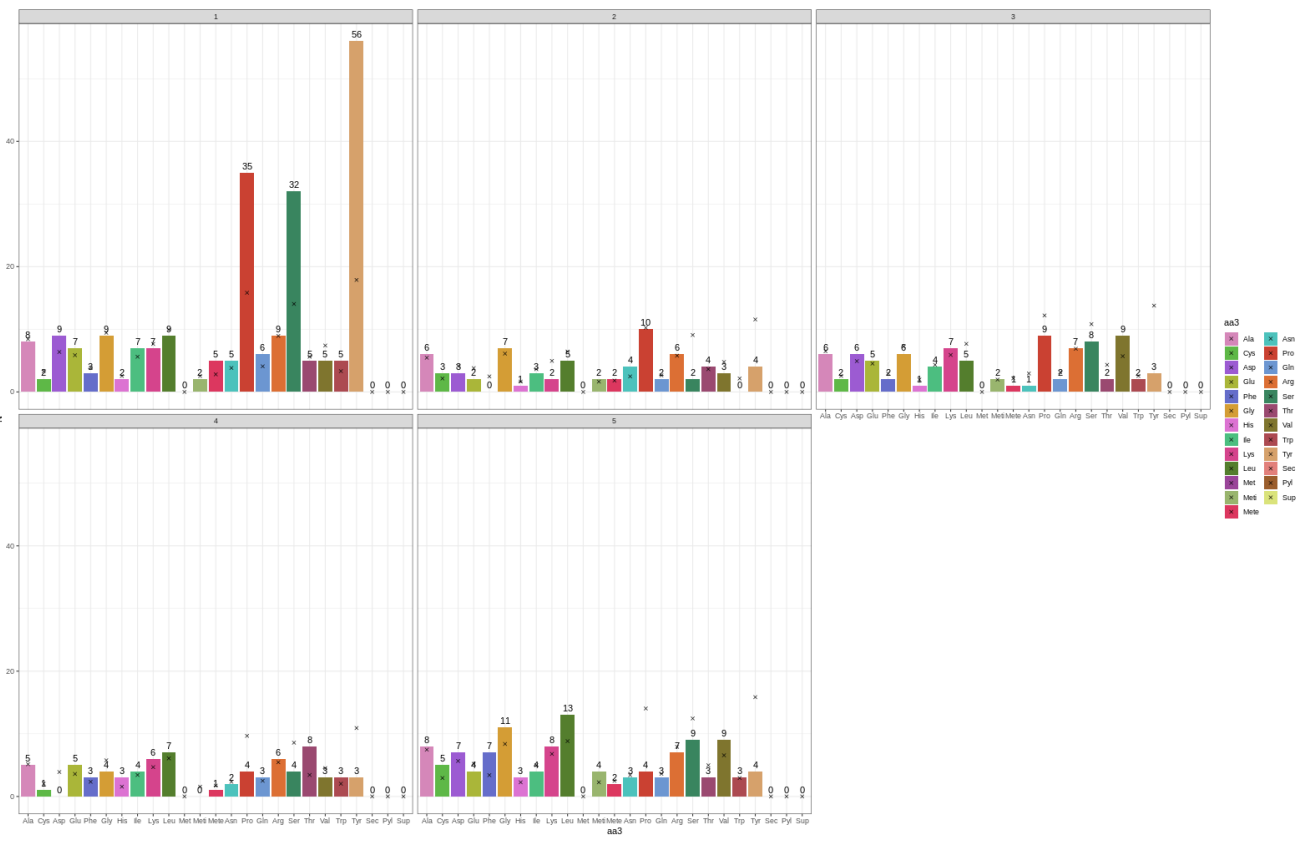

Heatmap of observed vs. expected counts per chromosome

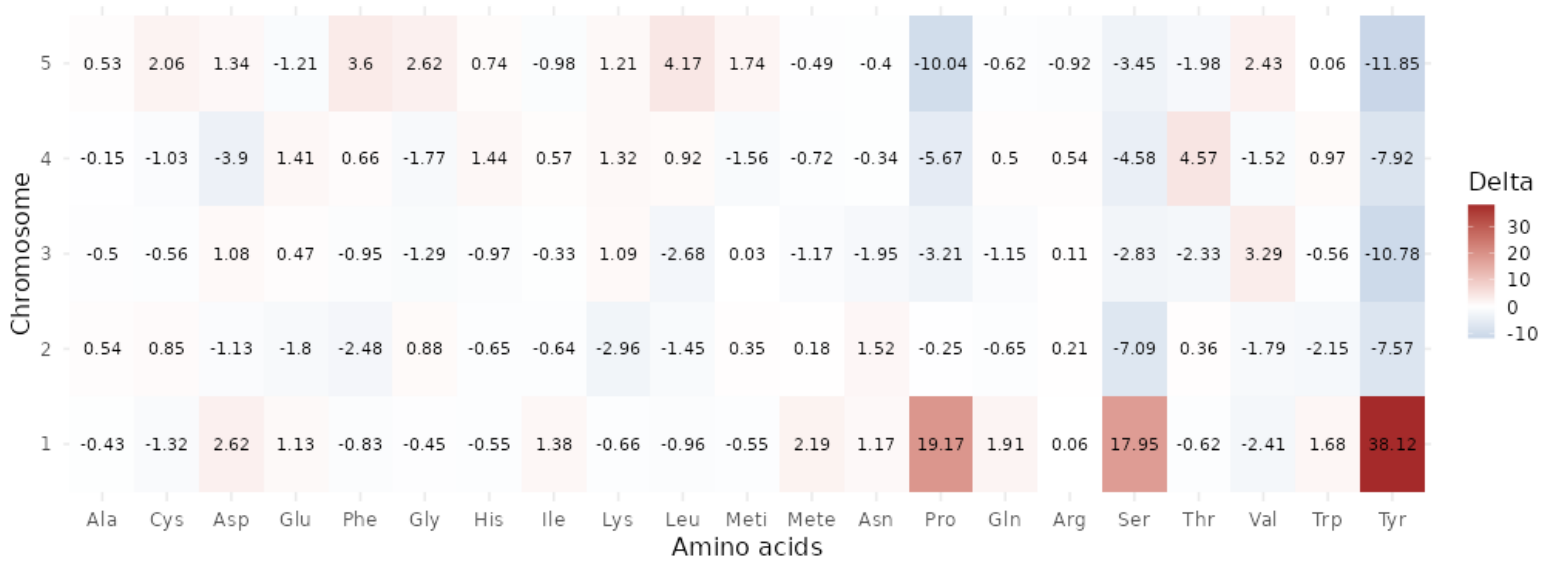

tDNA Pairwise

Consecutive tDNA pair frequency heatmap

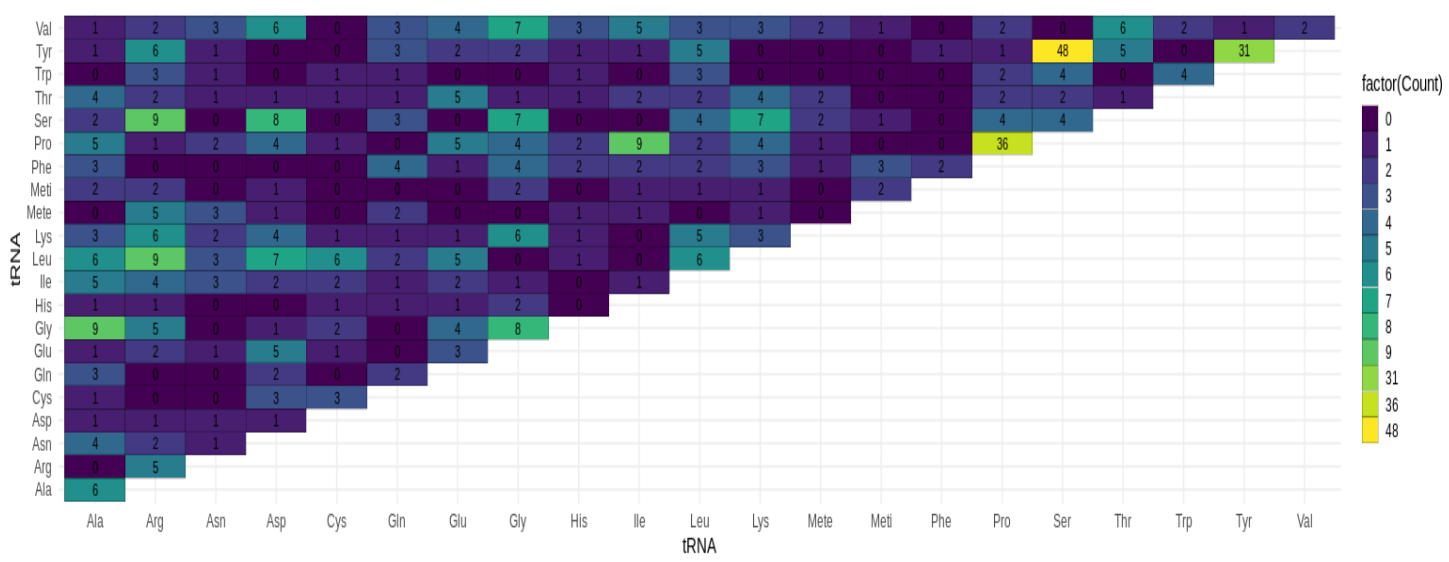

tDNA adjacency distances boxplot

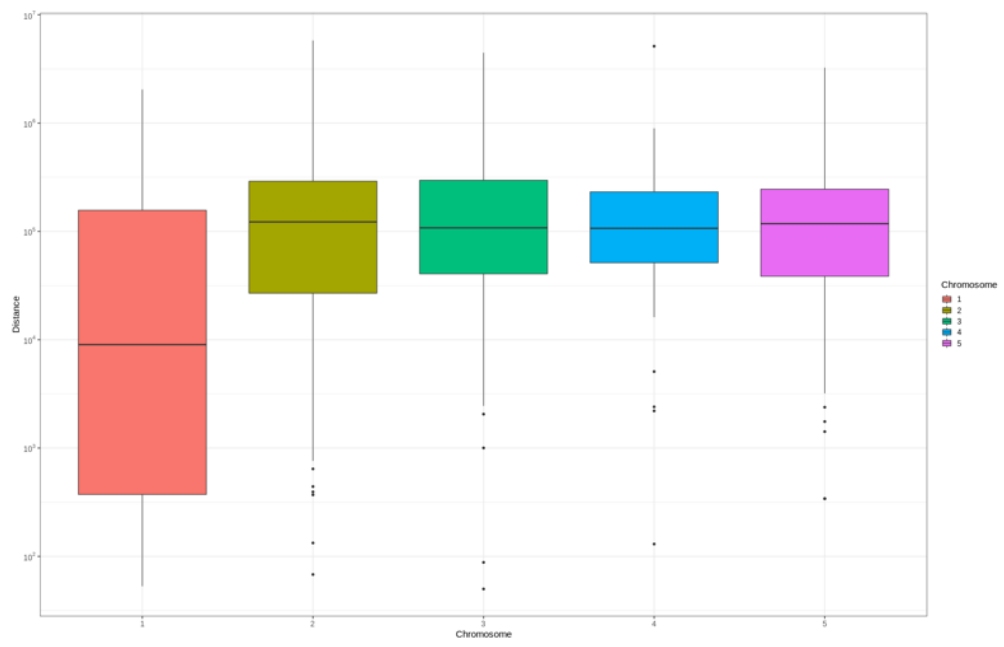

tDNA adjacency distances point

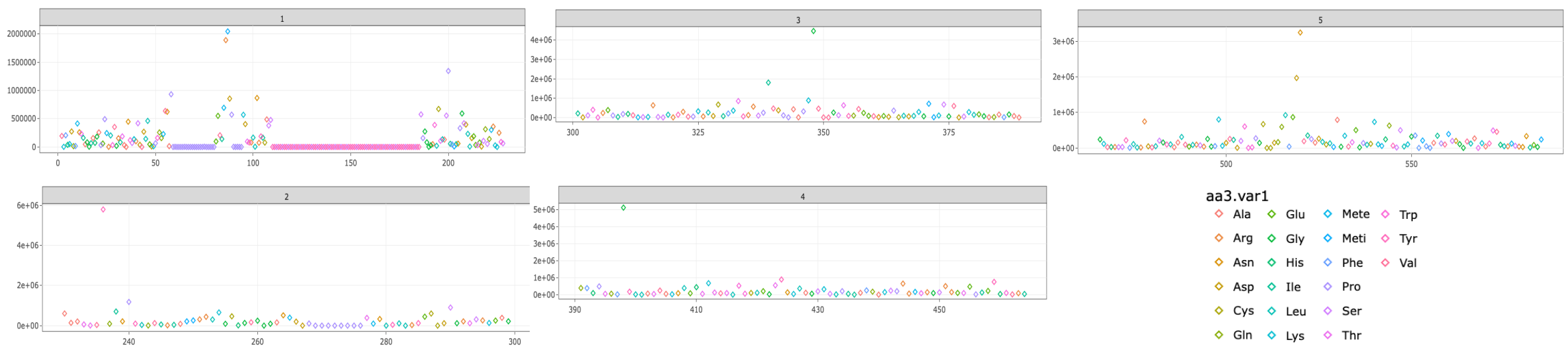

tDNA Clusters

Cluster plot

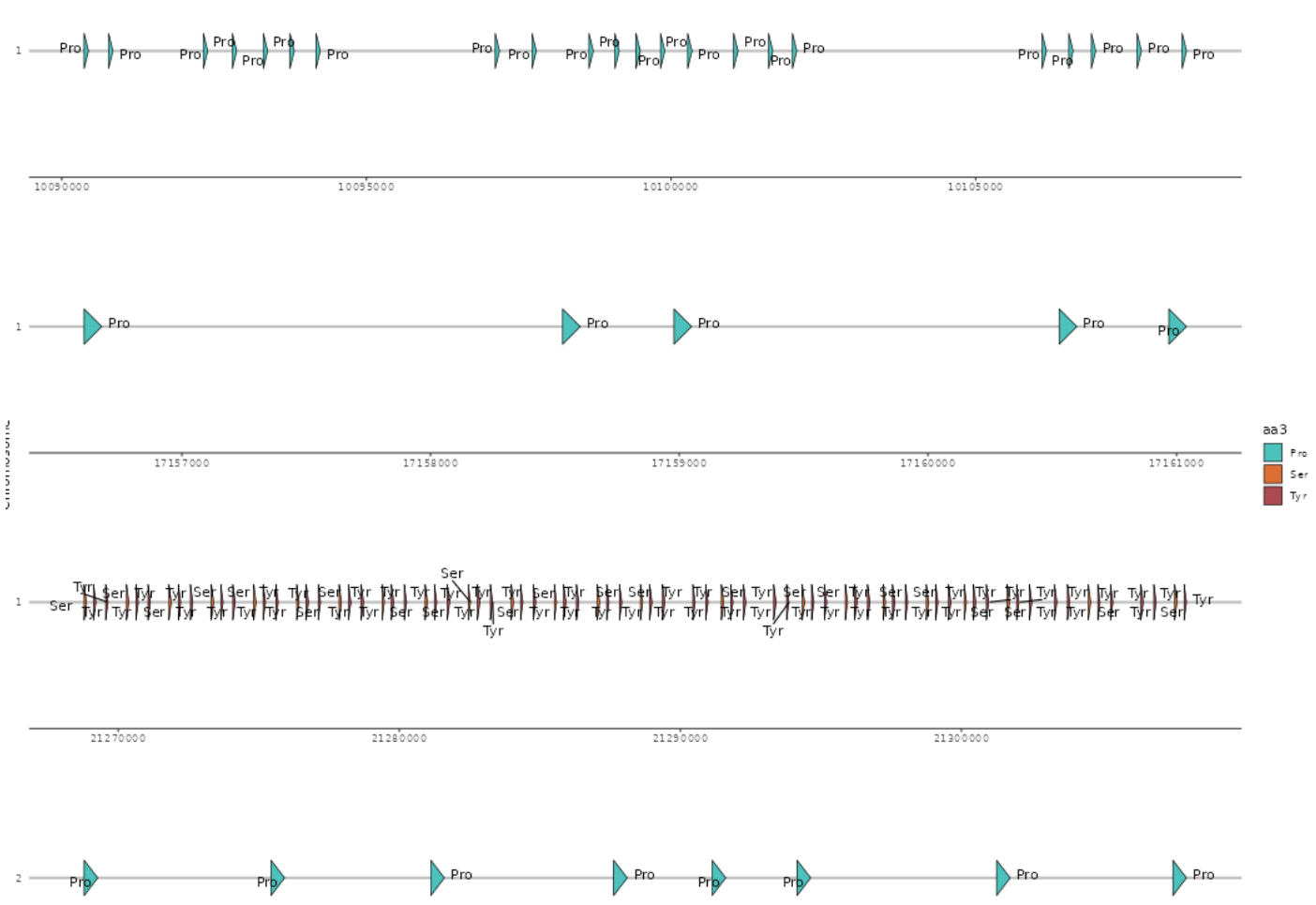

Centromere Positioning

Chromosomes with estimated centromere regions

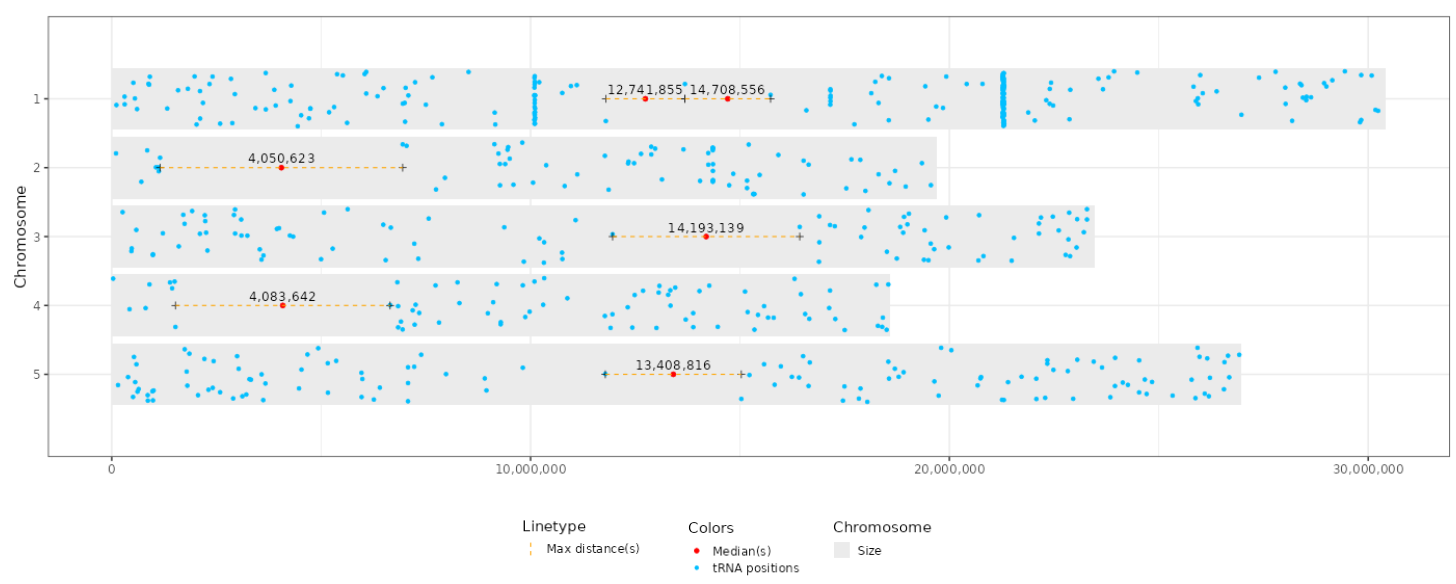

Supplemental Figure S1. Examples of data analysis performed on the ShinytRNA platform for *Arabidopsis thaliana* (Ath) (https://nebula.ibmp.unistra.fr/shinytRNA/).

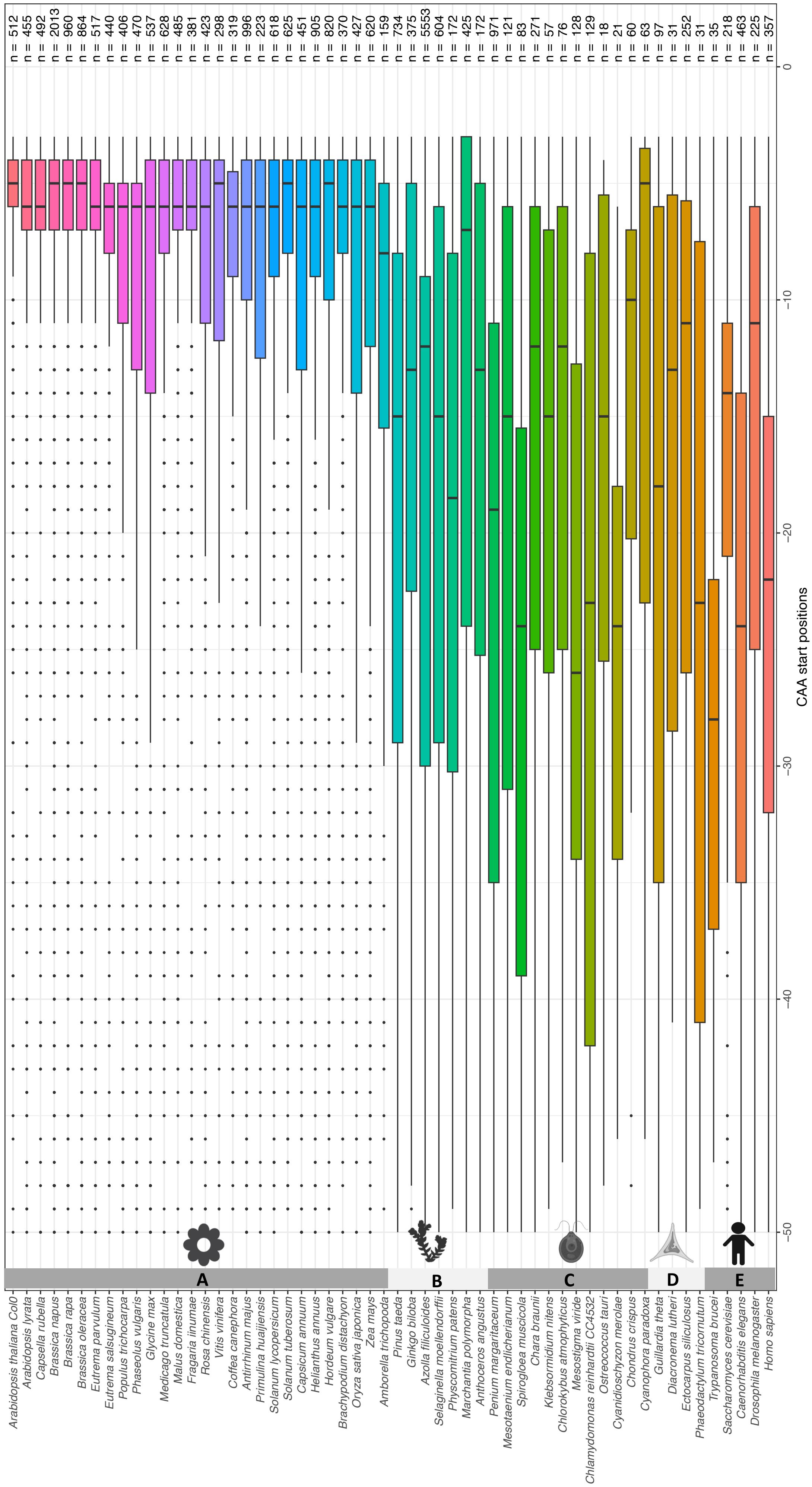

Figure S2. Boxplot showing the CAA start position of the first CAA motif in the region upstream of the 5′ end of tDNAs. (**A**) Angiosperms, (**B**) other embryophytes excluding angiosperms, (**C**) other Archaeplastida, (**D**) photosynthetic organisms with secondary plastids, and (**E**) non-photosynthetic organisms. n = number of analyzed tDNAs.

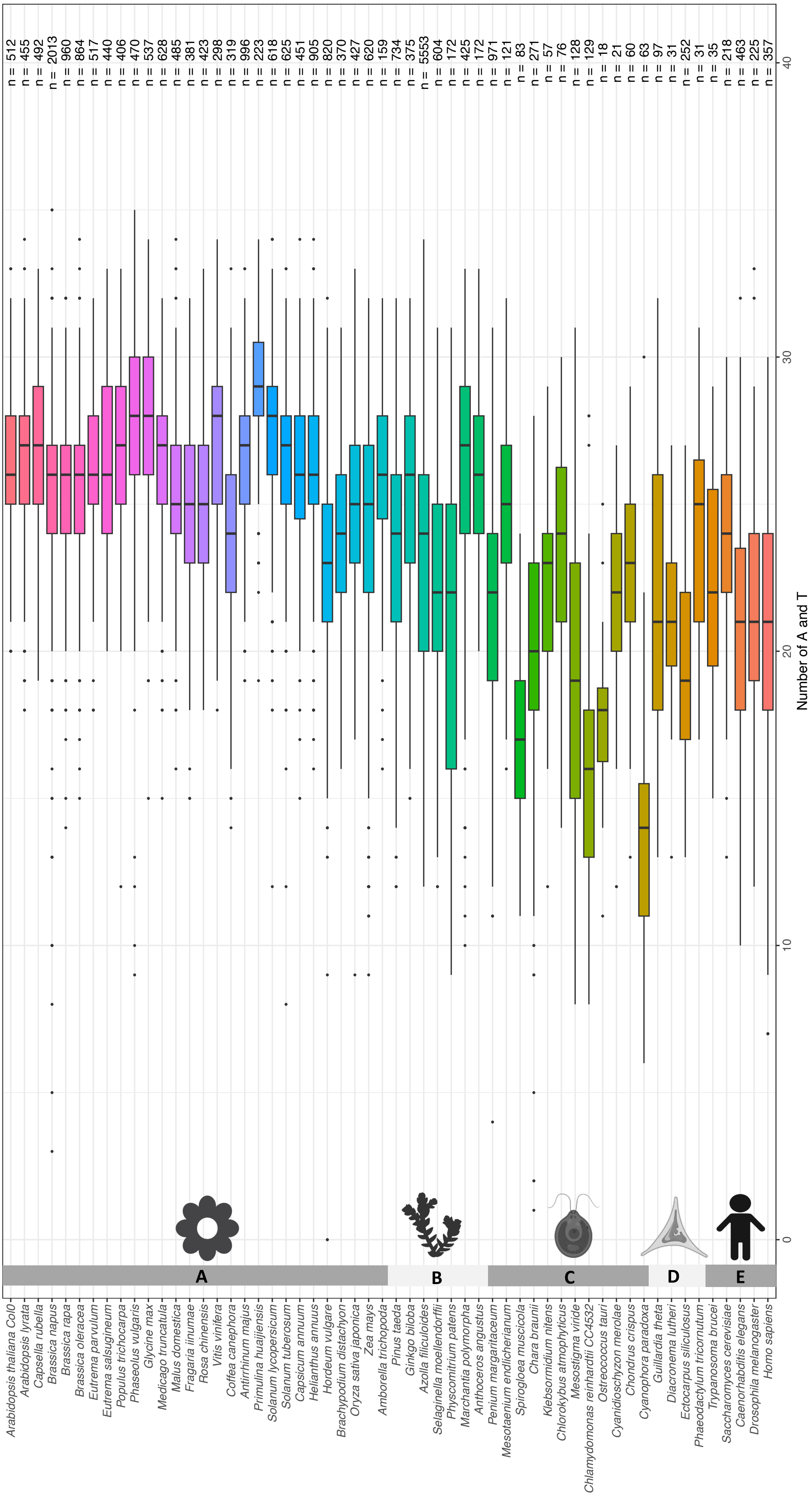

Figure S3. Boxplot showing the AT content in the region from –50 to –35 bp upstream of the 5′ end of tDNAs. (**A**) Angiosperms, (**B**) other embryophytes excluding angiosperms, (**C**) other Archaeplastida, (**D**) photosynthetic organisms with secondary plastids, and (**E**) non-photosynthetic organisms. n = number of analyzed tDNAs.

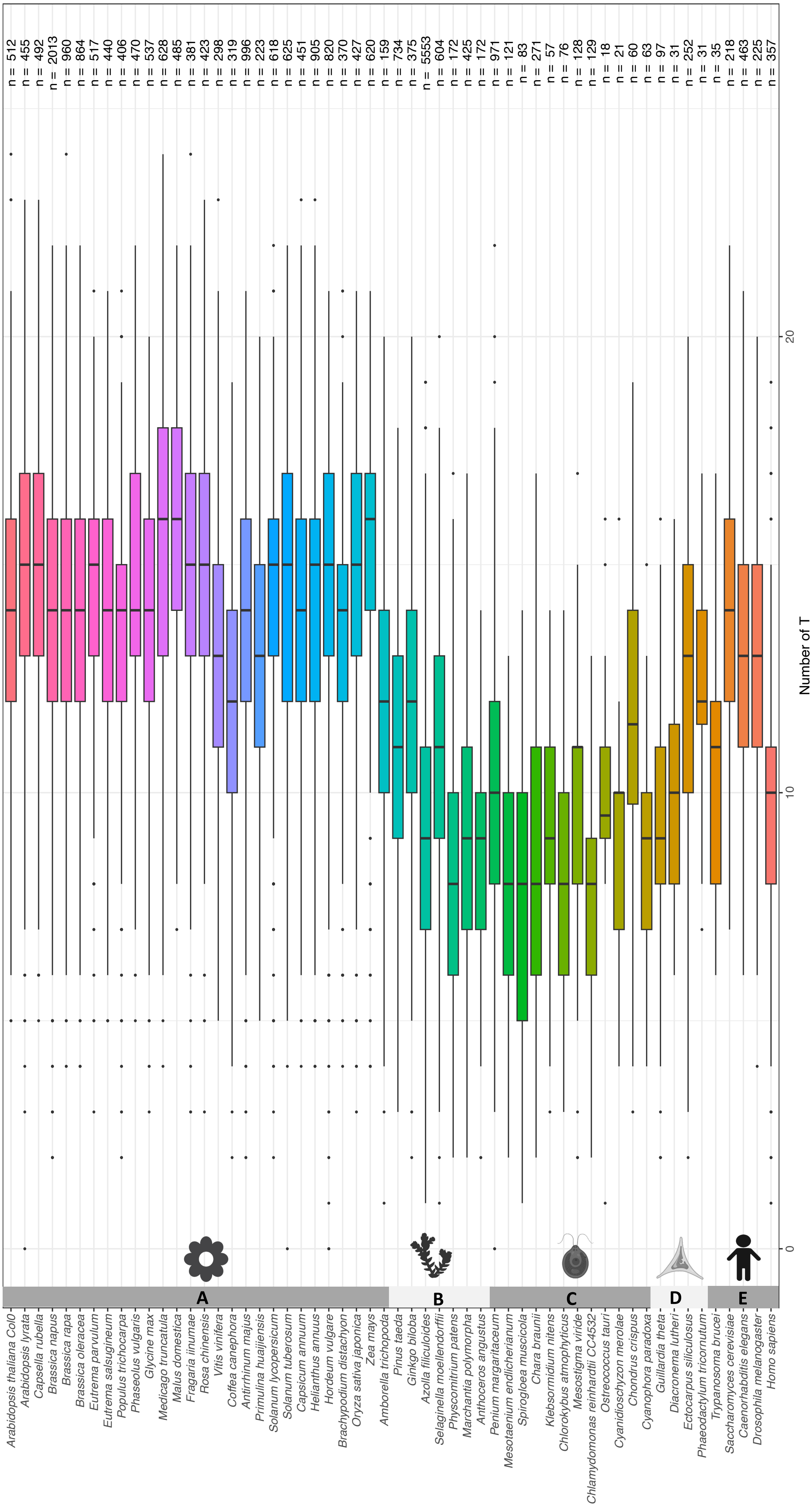

Figure S4. Boxplot showing the T content within 25 bp downstream of the 3′ end of tDNAs. (**A**) Angiosperms, (**B**) other embryophytes excluding angiosperms, (**C**) other Archaeplastida, (**D**) photosynthetic organisms with secondary plastids, and (**E**) non-photosynthetic organisms. n = number of analyzed tDNAs.

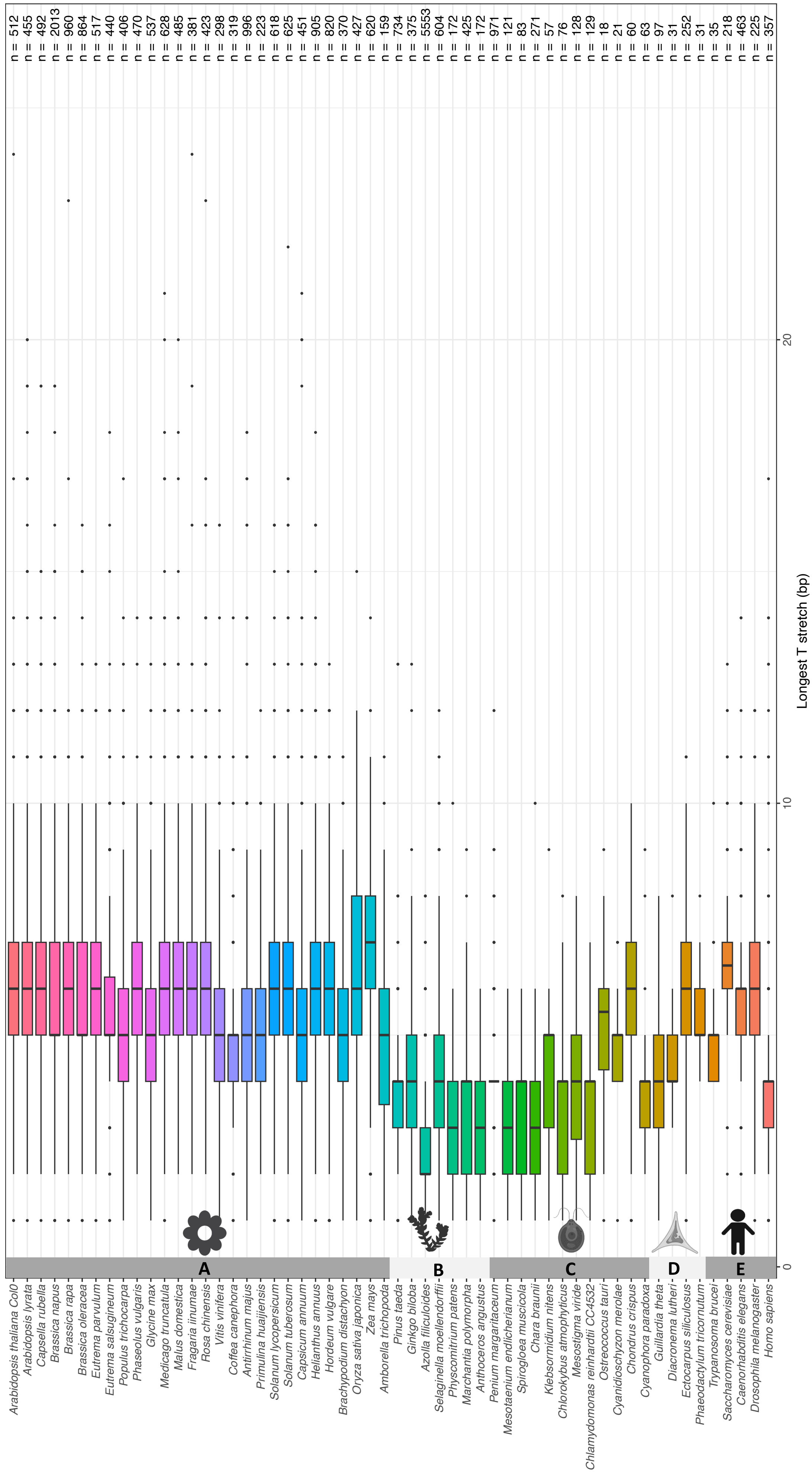

Figure S5. Boxplot showing the length of the longest T stretch located within 25 bp downstream of the 3′ end of tDNAs. (**A**) Angiosperms, (**B**) other embryophytes excluding angiosperms, (**C**) other Archaeplastida, (**D**) photosynthetic organisms with secondary plastids, and (**E**) non-photosynthetic organisms. n = number of analyzed tDNAs.

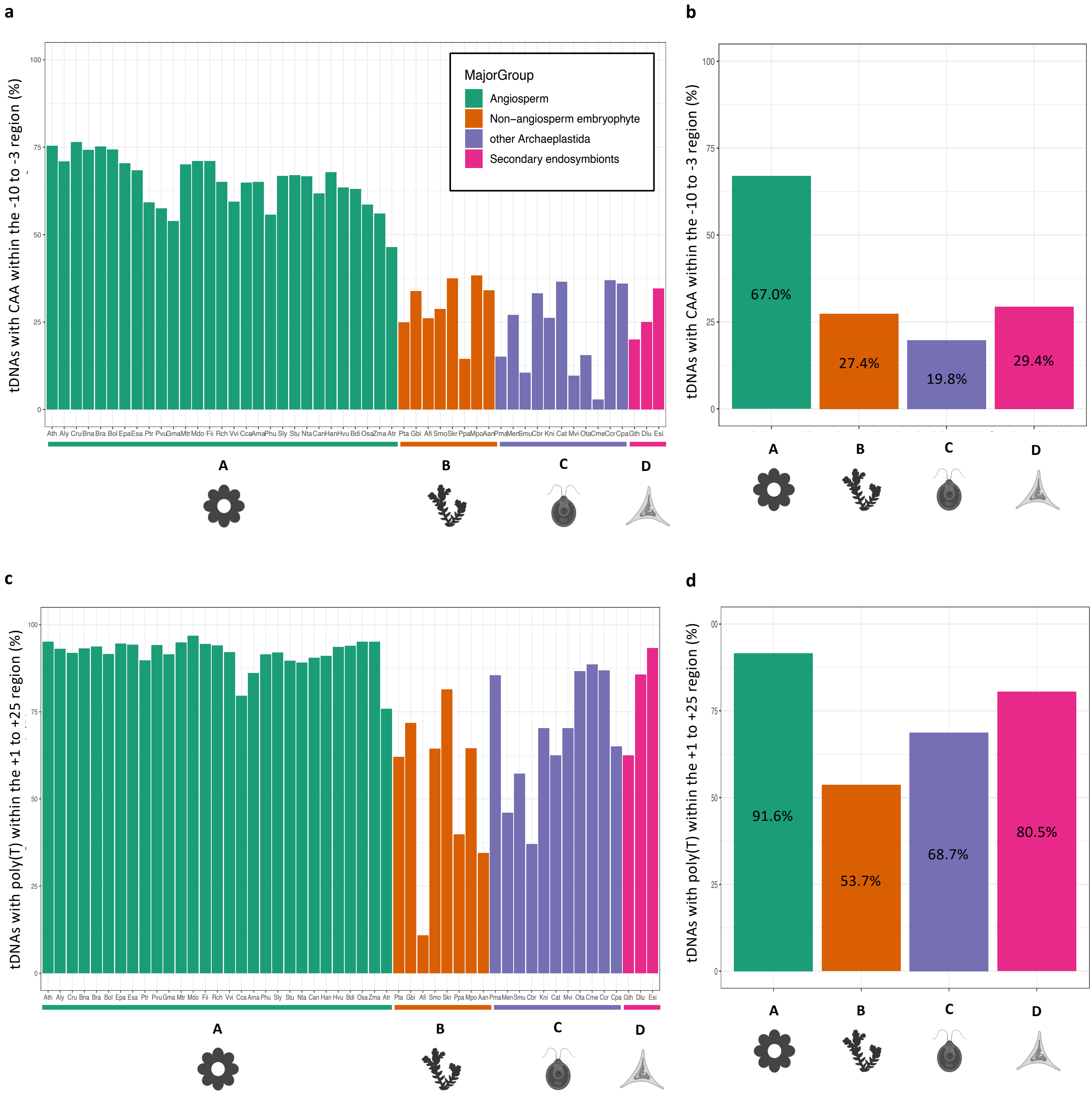

Figure S6. Histograms showing the proportion of tDNAs with a CAA motif within the −10 to −3 upstream region in photosynthetic species (**a**), and in major photosynthetic groups (**b**); and the proportion of tDNAs harboring at least one poly(T) stretch of ≥4 consecutive T residues within the +1 to +25 downstream region in photosynthetic species (**c**), and in major photosynthetic groups (**d**). In panels **b** and **d**, proportions are calculated globally across all tDNAs within each group. (A) Angiosperms, (B) other embryophytes excluding angiosperms, (C) other Archaeplastida, (D) photosynthetic organisms with secondary plastids.

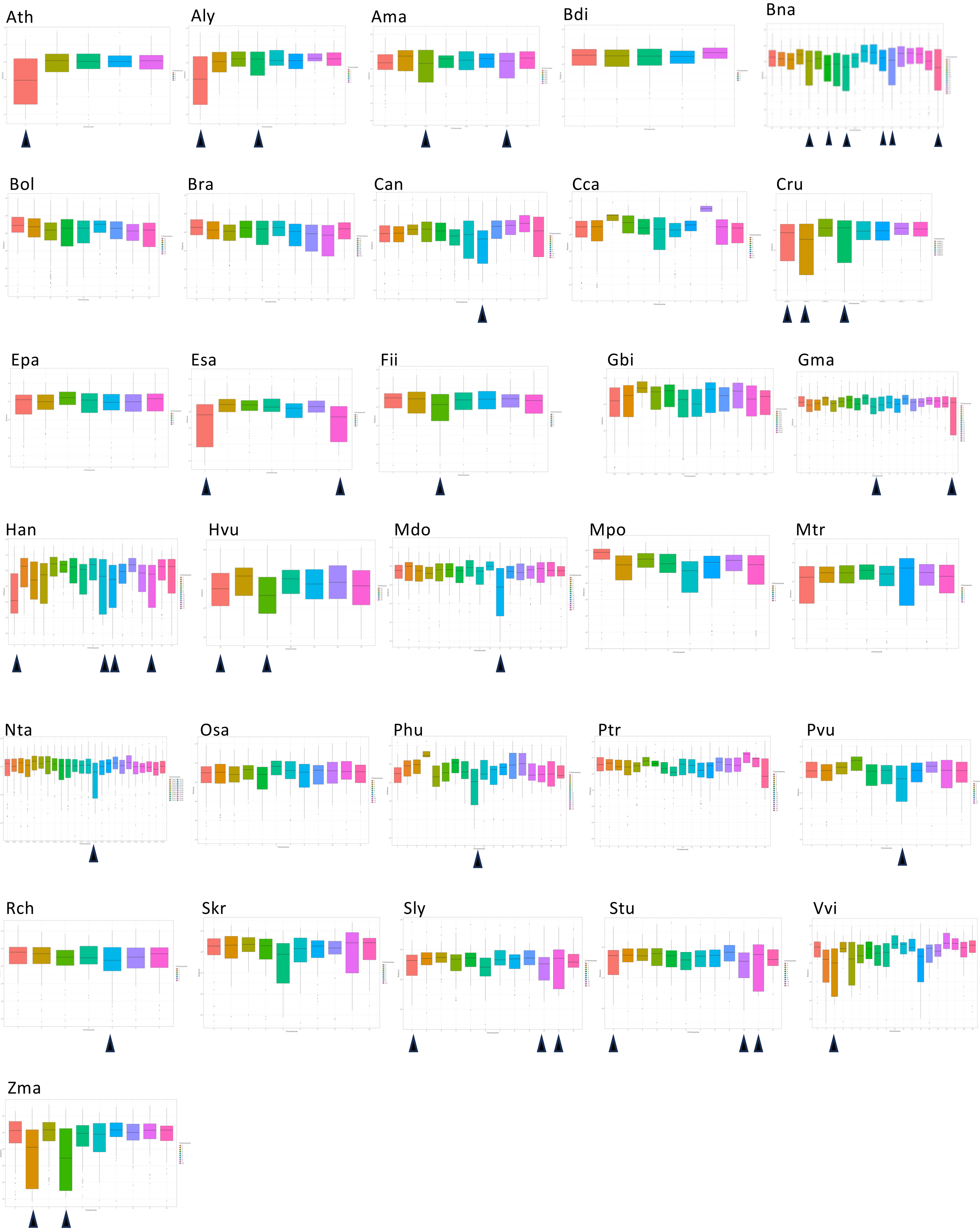

Figure S7. Boxplots showing the distance (d) between consecutive tDNAs on chromosomes from land plant species. Arrow heads indicate the main clusters on the respective chromosomes. Names of organisms are abbreviated as in Table S1. All boxplots are available at https://nebula.ibmp.unistra.fr/shinytRNA/.

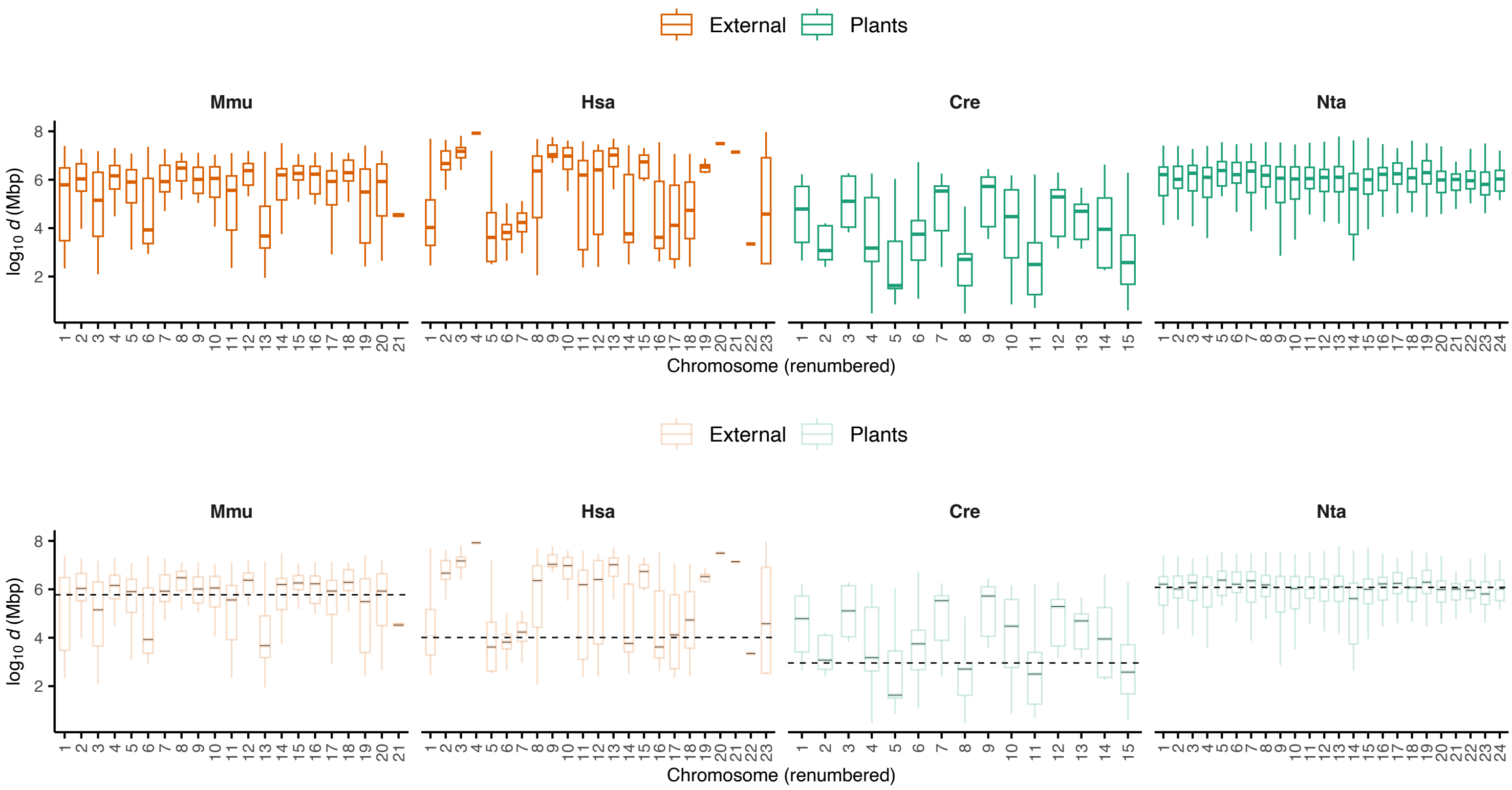

Figure S8: Comparison of tDNA organization in *Chlamydomonas reinhardtii* (Cre) versus a representative plant species *Nicotiana tabacum* (Nta) and two non-plant species *Mus musculus* (Mmu) and *Homo sapiens* (Hsa). Boxplots display the distribution of intrachromosomal intergenic distances between tDNAs ($\log_{10} d$, in Mbp). Horizontal dashed lines indicate median intergenic distance between consecutive tDNAs.

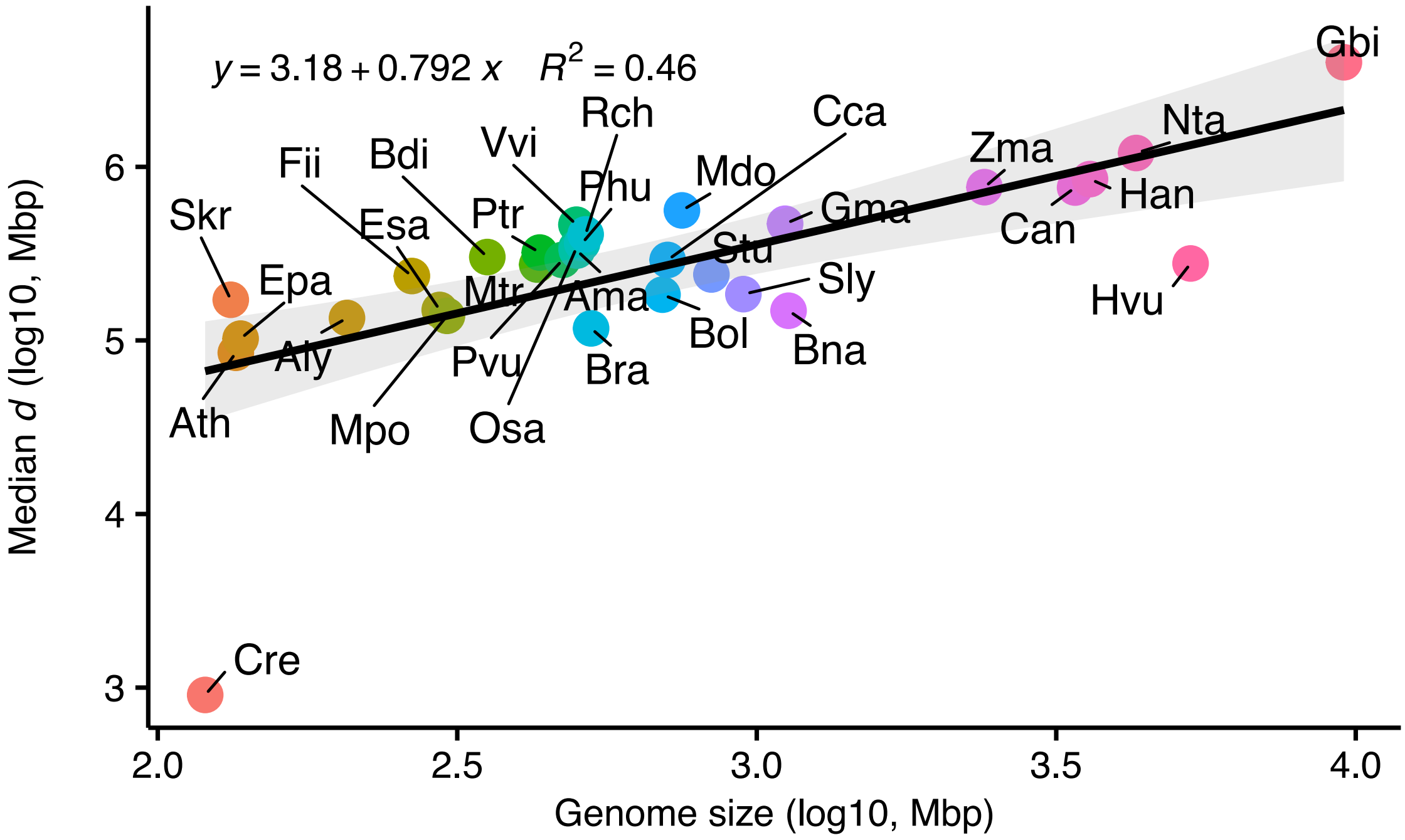

Figure S9. tDNA organization in the green lineage including *Chlamydomonas reinhardtii*. The representation is identical to Figure 4c but including *C. reinhardtii* (Cre).

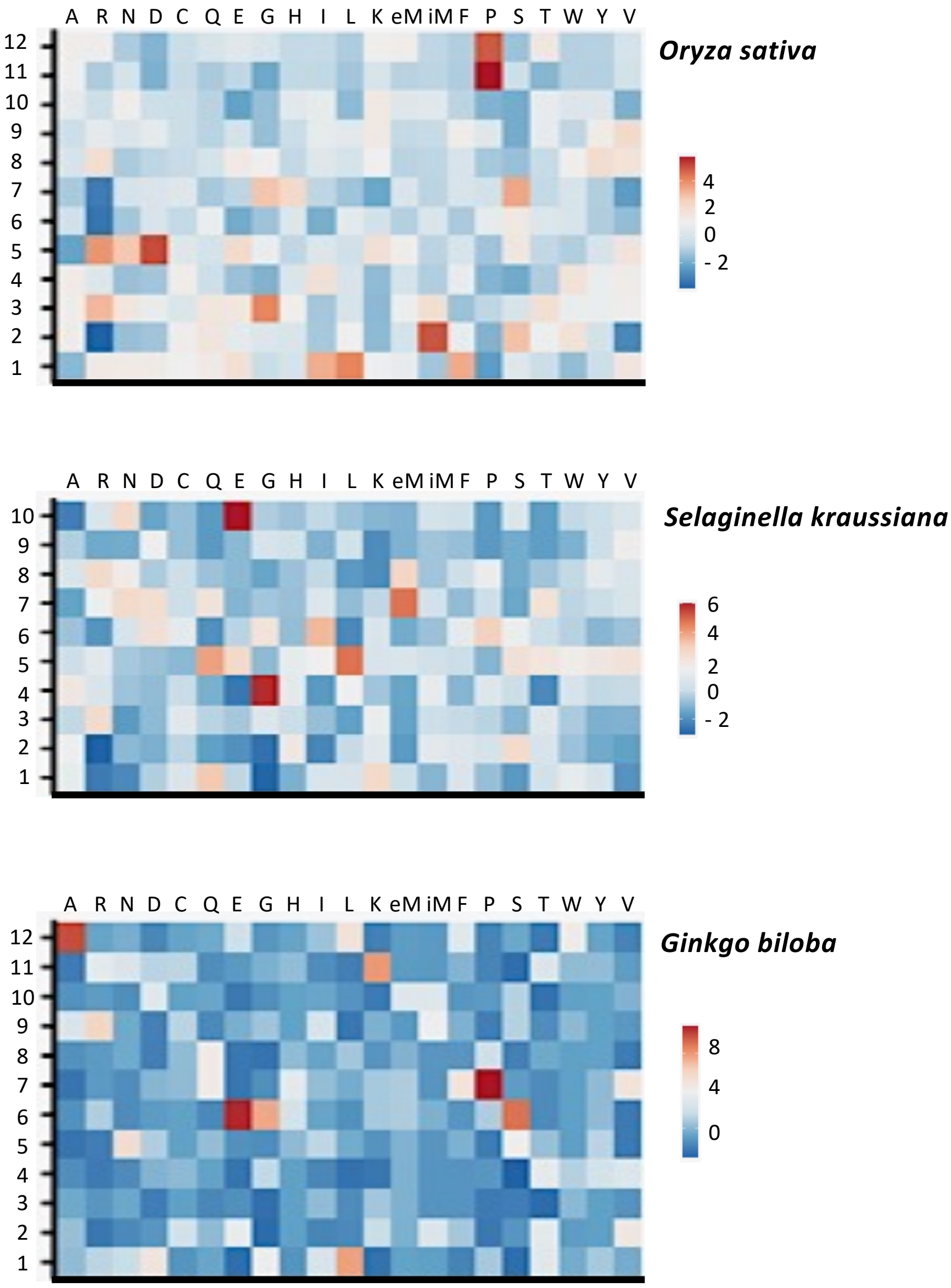

Supplemental Figure S10. Heatmaps showing the chromosomal distribution of isoacceptor tDNA families in three species (*Oryza sativa*, *Selaginella kraussiana*, and *Ginkgo biloba*). Rows correspond to chromosomes, columns to amino acid families (single-letter code). Colors indicate deviation from the expected random distribution normalized by genome size, with red denoting enrichment and blue depletion.

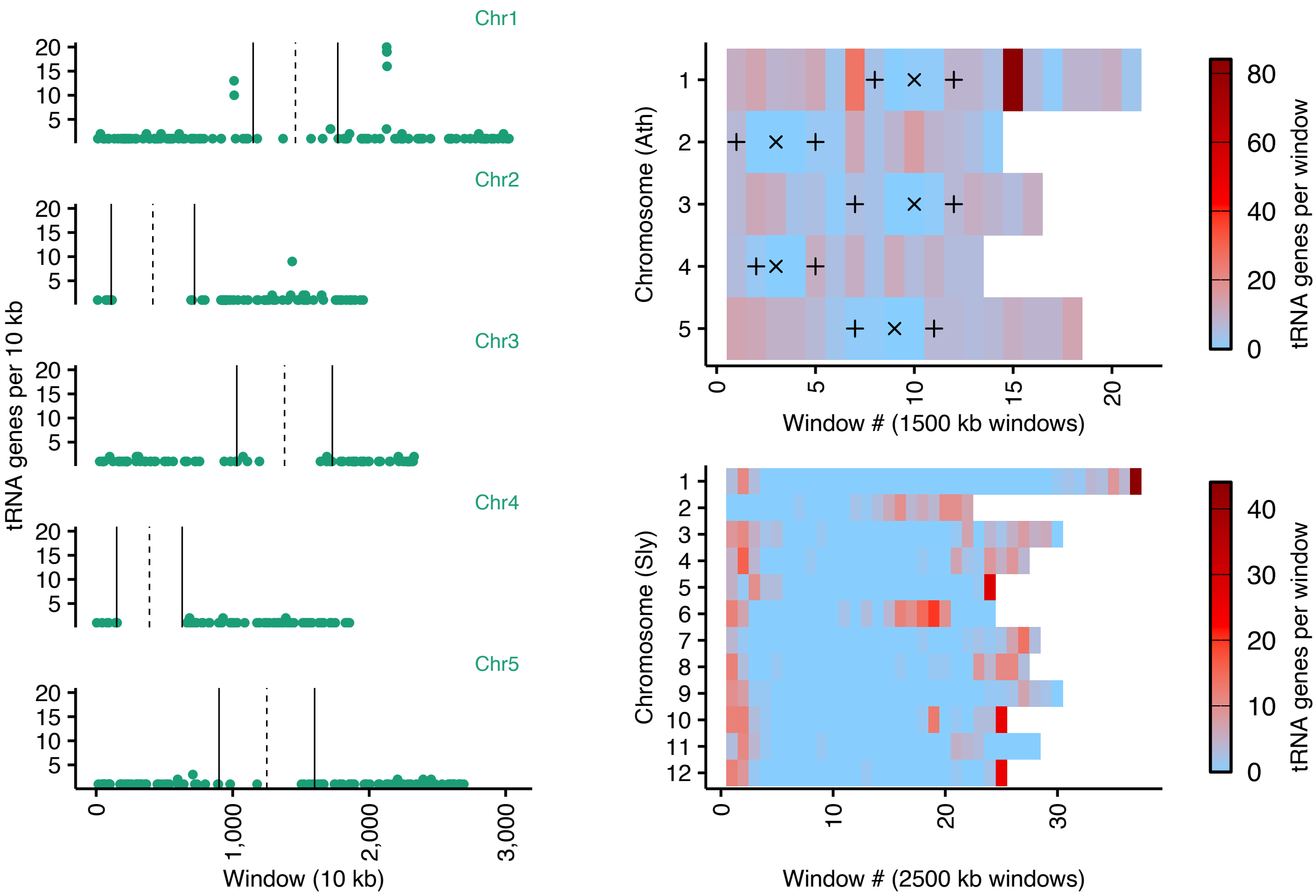

Supplemental Figure S11. Chromosomal distribution of tDNAs reveals large-scale genomic organization in plants. **A**) Number of tDNAs within consecutive 10-kb windows along the chromosomes of *Arabidopsis thaliana*. Solid and dotted vertical black lines indicate the boundaries and central coordinates of centromeric regions, respectively. **B**) Heatmap of tDNA density across the chromosomes of *A. thaliana* and *Solanum lycopersicum*. The (+) and (×) symbols mark the approximate centromeric regions and their central coordinates in *A. thaliana*, respectively.

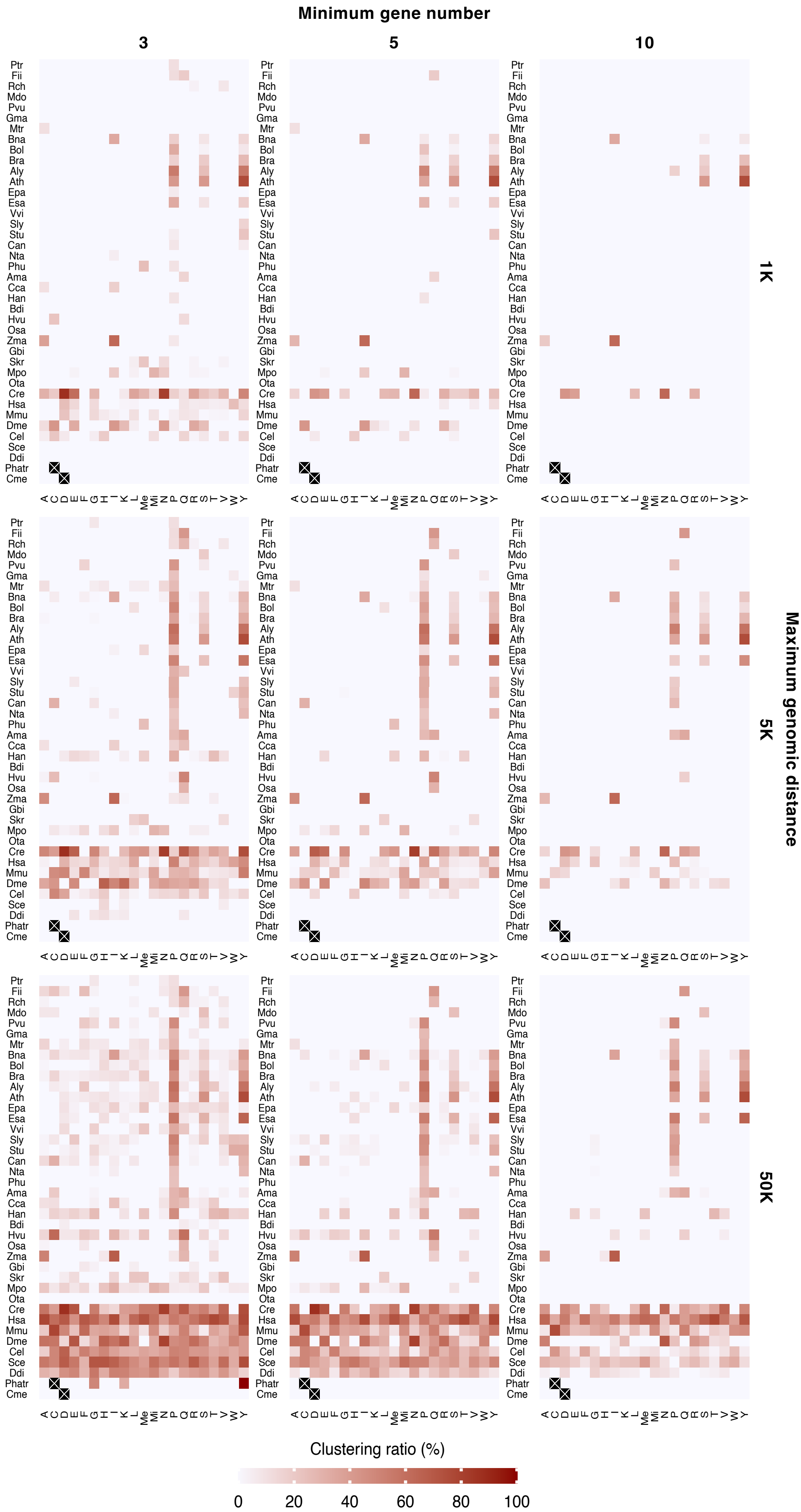

Supplemental Figure S12. tDNA cluster identification upon different filtering conditions. White indicates no clustering (all genes isolated), while dark red indicates full clustering (all genes grouped). In *Cyanydoschyzon merolae* (Cme) and *Phaelodactylum tricornutum* (Phatr), asparagine (D) and cysteine (C) tDNAs could not be confidently annotated and were therefore excluded (black crossed-out regions). Species labels identical to Table S1.

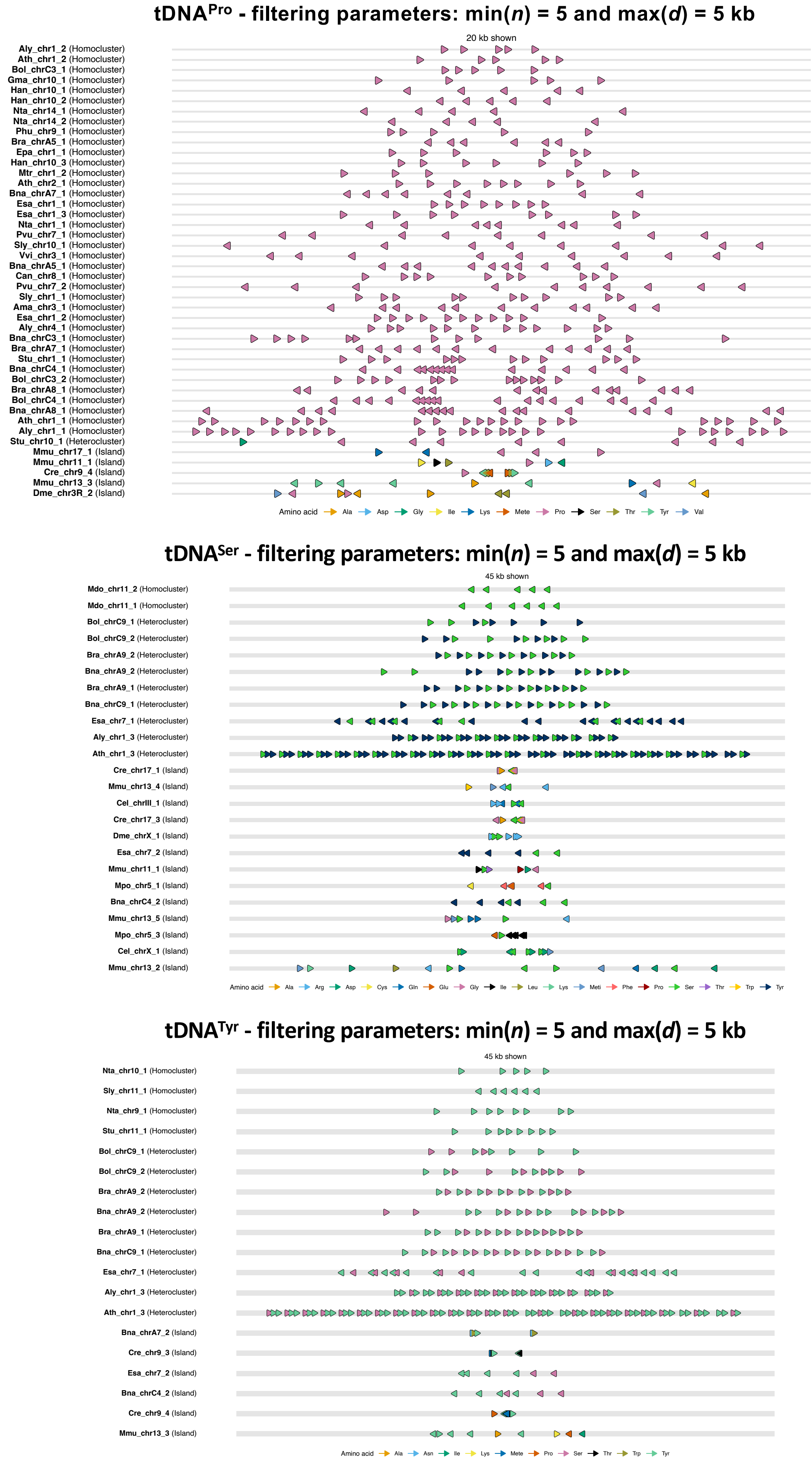

Supplemental Figure S13. Identification of proline-, serine-, and tyrosine-containing tDNA clusters using ShinytRNA. Representative genomic clusters containing at least 5 $tDNA^{Pro}$ (top), $tDNA^{Ser}$ (middle), or $tDNA^{Tyr}$ (bottom) within ≤5 kb. Each horizontal line represents an individual genomic cluster, ordered by species and chromosome. Colored arrowheads indicate individual tDNA loci and their associated amino acid isotypes according to the color code shown below each panel. Arrow orientation indicates the direction of transcription.
