# Supplementary material for: Multilevel genomic constraints shape nuclear tRNA gene organization in plants
Source: Plant J. 2026 Aug 4;127(3):e71075. doi: 10.1111/tpj.71075 (PMC13437025; doi:10.1111/tpj.71075)
Supplement: Supplementary file 2 [file TPJ-127-0-s004.docx]

**Supplemental Methods**

**Detailed computational procedures**

The analytical framework implemented in this study was designed to quantify nuclear tDNA organization across genomic scales, including intergenic spacing, chromosome-level distribution, adjacency relationships, local clustering, and cis-regulatory sequence conservation. Detailed formulas, threshold definitions, and algorithmic procedures used for each analysis are provided below.

**Inter-tDNA distance calculation**

Inter-tDNA distance (d) was defined as the genomic distance between two consecutive tRNA genes (*trnᵢ* and *trnᵢ₊₁*) along a chromosome:

*d* = *start* (*trn_i+1_*) – *end* (*trn_i_*)

Distances were computed for all adjacent pairs within each chromosome. When indicated, loci belonging to clusters were excluded prior to distance calculation to estimate background dispersion. Distance distributions were summarized using median, mean, standard deviation, and extrema. For visualization, values were log10-transformed.

**Chromosome-level expected distribution model**

To assess whether tRNA gene isotypes are randomly distributed across chromosomes, expected counts were calculated assuming proportionality to chromosome length:

$$E_{i,a}=\frac{N_{a}}{L_{genome}}\times L_{i}$$

where: $E_{i,a}$ = expected number of tRNA genes of isotype *a* on chromosome *i* ; $N_{a}$ = expected number of tRNA genes of isotype *a* on chromosome *I* ; $L_{i}$ = length of chromosome *I* ; and $L_{genome}$ = total genome size

Deviation from expectation was quantified using standardized residuals:

$$R_{i,a}=\frac{O_{i,a}-E_{i,a}}{\sqrt{E_{i,a}}}$$

where *Oi,a* is the observed count.

Significance of deviations from expected distributions was assessed using chi-square tests with 1 degree of freedom ($p<0.05;$ χ² > 3.84). Isotypes were classified as over-represented, under-represented, or neutral based on fold-change and statistical significance. In addition, a global non-randomness test was performed per isotype by aggregating chromosome-level chi-square statistics.

**Adjacency and pairwise isotype analysis**

To evaluate non-random organization of neighbouring tRNA genes, pairwise adjacency frequencies were computed based on ordered loci along chromosomes.

Expected frequencies under a random model were calculated as:

For identical isotypes (a = b):

$$E_{a,a}=\frac{N_{a}(N_{a}-1)}{N(N-1)}\times T$$

For distinct isotypes (a ≠ b):

$$E_{a,b}=\frac{2N_{a}N_{b}}{N(N-1)}\times T$$

where: $N_{a}$and $N_{b}$ = counts of isotype *a* and *b* ; $N$= total number of tRNA genes ; $T$= total number of adjacent pairs.

For each pair, the following metrics were computed:

- observed and expected counts
- observed and expected frequencies (%)
- fold enrichment
- frequency deviation (Δ)
- chi-square statistic and associated p-value

Observed and expected values were compared using chi-square tests with 1 degree of freedom.

**Centromere position approximation**

Putative centromeric regions were approximated based on large inter-tDNA distances. For each chromosome, the largest intergenic intervals were identified, and midpoint positions between adjacent loci were calculated as:

$$\text{C}=\text{end}_{n-1}+\frac{d}{2}$$

These regions were interpreted as low-density genomic intervals potentially corresponding to centromeric regions. When experimentally validated centromere coordinates were unavailable, putative centromeric regions were approximated from the largest inter-tDNA intervals. These proxy regions were used to assess large-scale tDNA exclusion patterns and should be interpreted cautiously.
